# An Integrated Meta-QTL and Transcriptome Analysis Provides Candidate Genes Associated with Drought Tolerance in Rice Seedlings

**DOI:** 10.3390/plants14233645

**Published:** 2025-11-29

**Authors:** Yinji Jin, Weize Dou, Tianhao Wang, Zhuo Jin, Songquan Wu

**Affiliations:** College of Agriculture, Yanbian University, Yanji 133002, China; yinjij0116@163.com (Y.J.); 18843900626@163.com (W.D.);

**Keywords:** rice, drought tolerance, meta-QTL, RNA-seq

## Abstract

Drought stress, intensified by climate change, poses a significant threat to global rice security. To identify stable quantitative trait loci (QTL) associated with drought tolerance in rice under different genetic backgrounds and environmental conditions, this study combined 901 drought-tolerant QTLs reported in 52 independent studies published between 2000 and 2023, which were subsequently meta-analyzed and condensed into 77 meta-QTLs (MQTLs). Among them, 23 MQTLs were validated in seven independent genome-wide association studies (GWAS) on drought tolerance in rice, each conducted using different natural populations. The confidence intervals (CIs) of the MQTLs were substantially narrowed, with the reduction factor ranging from 2.44 to 20.40 relative to the original QTLs. To further explore key genes for drought tolerance, we screened for genes located within the MQTL regions and differentially expressed in our RNA-seq data, yielding 3851 drought-responsive differentially expressed genes (DEGs). These DEGs were then subjected to a refinement process that included Mfuzz clustering, cis-regulatory element (CRE) analysis, protein–protein interaction (PPI) network analysis and AlphaFold-based structural modeling of their encoded proteins. This stepwise filtering identified eleven drought-responsive hub proteins, nine with annotated functions and two functionally uncharacterized. Following further prioritization, *LOC_Os04g35340* and *Os07g0141400* were established as core candidate genes (CGs) for dissecting the genetic and biochemical basis of drought tolerance in rice.

## 1. Introduction

Rice (*Oryza sativa* L.) is a staple food for nearly half of the global population, underpinning food security and sustainable agricultural development [1]. However, the goal of increasing annual production by 44 million tonnes by 2050 [2] is severely threatened by intensifying drought, a consequence of climate change that risks impacting over 50% of arable land [3]. This challenge is particularly acute for rice, a semi-aquatic crop highly sensitive to water deficits [4]. Consequently, understanding and enhancing drought tolerance has become a paramount objective in rice breeding.

Enhancing drought tolerance is crucial for stabilizing rice yield under water deficit [5]. However, drought tolerance is notoriously complex, controlled by multiple genes and involving physiological processes such as root development, osmotic adjustment, and hormone signal transduction [6]. This complexity, combined with its low heritability due to minor polygene effects and strong environmental influence [7], makes it highly inefficient to improve through traditional breeding methods. Hence, analytical methods capable of distinguishing genetic effects from environmental noise are indispensable for improving drought tolerance and guiding precision breeding. The advent of genomic technologies, such as molecular markers, has provided a powerful solution in this regard. These tools enable the mapping of quantitative trait loci (QTLs) in well-controlled experiments or through multi-environment trials, allowing for the control of environmental variance. This has become a fundamental strategy for identifying genes underlying drought tolerance [8]. Consequently, numerous QTLs conferring drought tolerance have been identified, laying a solid foundation for gene discovery and molecular breeding [9].

Meta-QTL (MQTL) analysis has emerged as a pivotal approach for dissecting complex traits [10]. Its unique advantage lies in synthesizing QTL data from diverse genetic backgrounds and environments to identify stable genomic regions with significantly refined confidence intervals (CIs), thereby dramatically improving the precision of candidate gene identification [11,12]. The robustness of MQTLs is well-established across numerous crop species for a variety of agronomically important traits, such as yield [13], salt tolerance [14], cold tolerance [15], and drought tolerance [16]. To further enhance the power of MQTLs, they can be integrated with independent genomic approaches. For instance, genome-wide association studies (GWAS) identify relevant genetic loci for complex traits by detecting statistical associations between genomic variants and phenotypic variation. [17]. The integration of MQTL and GWAS results thus provides robust mutual validation and effectively elucidates the underlying genetic mechanisms. [11]. Moreover, the integration of RNA-seq data provides a powerful screening tool to identify key candidate genes within MQTL regions based on their expression patterns, significantly enhancing the efficiency of candidate gene screening [18].

In the present study, we first systematically collated rice drought-tolerant QTLs reported over the past two decades and conducted a comprehensive meta-QTL analysis. The core objective was to identify stable MQTLs, reduce their CIs, and finely map the genetic regions associated with drought tolerance in rice. Second, we further utilized RNA-seq data from two specific materials—the wild-type and T608 mutant—to narrow down drought-related intervals within the identified MQTL regions. We analyzed the expression patterns of genes within the MQTL regions in these two materials and, through their transcriptional dynamic characteristics, screened for genes with drought-responsive expression characteristics, thereby more precisely delineating the functional intervals associated with drought tolerance.

## 2. Results

### 2.1. Collection of QTL Data Associated with Drought Tolerance in Rice from Previous Studies

**Figure 1:**
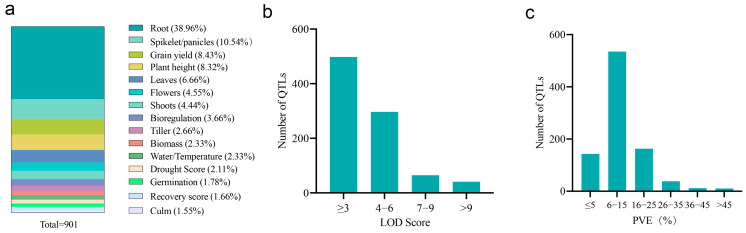
Characteristics of the drought-tolerant QTLs included in the meta-analysis. (**a**) Proportion of QTLs associated with trait categories. (**b**) Distribution of LOD scores for the initial QTLs. (**c**) Distribution of phenotypic variance explained (PVE) by the initial QTLs.

A total of fifty-two independent QTL studies, derived from crosses between drought-tolerant and drought-sensitive rice parents, were compiled for meta-analysis. These studies encompassed a wide range of genetic backgrounds (diverse varieties), population sizes, crossing methods, and environmental conditions. Combining these datasets revealed 901 drought-resistant QTLs, including those linked to multiple drought-responsive traits (Table 1). The QTL analysis focused on 15 distinct traits measured specifically under drought stress. Root architecture-related traits constituted the largest proportion (38.96%) of the collected QTLs, followed by spike/panicle traits (10.54%), yield (8.43%), and plant height (8.32%) (Figure 1a). Notably, QTLs detected under well-watered conditions showed minimal overlap in their genomic positions. The logarithm of the odds (LOD) scores and phenotypic variance explained (PVE) were compared with those detected under drought stress, confirming a strong genotype-by-environment interaction. Consequently, only drought-specific QTLs were retained for the subsequent meta-analysis. The LOD scores of these QTLs ranged from 1.54 to 39 (mean = 4.60) (Figure 1b), and the PVE by individual QTLs varied from 0.08% to 67.7% (Figure 1c).

### 2.2. Meta-Analysis of QTLs Conferring Drought Tolerance in Rice

**Figure 2:**
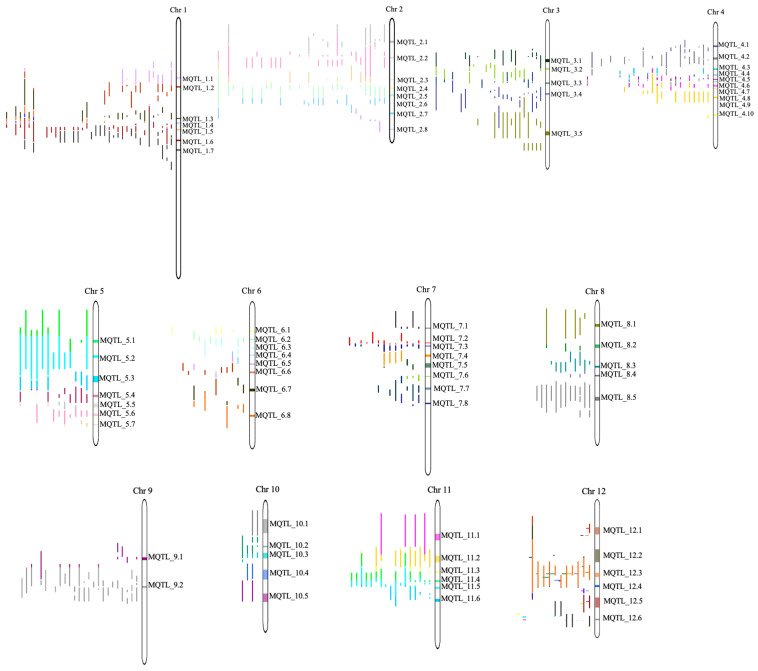
Chromosomal distribution of meta-QTLs for drought tolerance in rice. Chromosomes are displayed as vertical bars. Initial QTLs from individual studies are shown as colored segments (**left**), and the condensed meta-QTLs are indicated by colored blocks on the chromosomes.

**Figure 3:**
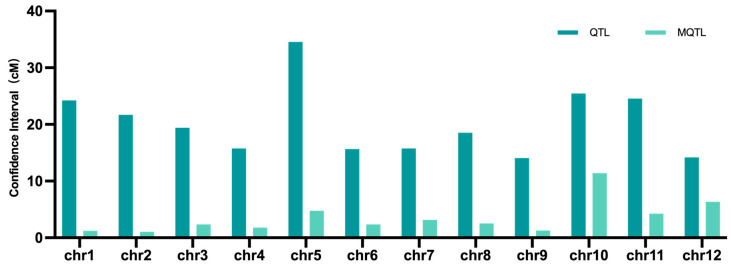
Comparison of average CI between initial QTLs and MQTLs on the 12 rice chromosomes.

A total of 77 consensus MQTLs were distilled from the 901 initial QTLs through a two-step analytical strategy: (i) chromosome-wise projection and clustering using BioMercator v4.2, followed by (ii) model selection across AIC, AICc, AIC3, BIC and AWE. These 77 MQTLs, named according to their chromosomal location, spanned physical intervals ranging from 0.9 Mb to 3.8 Mb, collectively covering approximately 138 Mb (≈36%) of the rice genome. Chromosome 4 contained the highest number of non-overlapping MQTLs (*n* = 10), whereas chromosome 9 harbored the fewest (*n* = 3) (Table A1) (Figure 2). Despite this uneven distribution (ranging from 3 on chromosome 9 to 10 on chromosome 4), MQTLs were identified on all 12 rice chromosomes, demonstrating the genome-wide genetic control of drought tolerance. The meta-analysis dramatically refined the QTL confidence intervals (CIs), reducing the average CI from 20.32 cM to 3.54 cM, which represents an 82.6% reduction (Figure 3). Chromosome 2 showed the sharpest compaction (20-fold), followed by chromosome 1 (19-fold), while the most modest reduction was observed on chromosome 9 (2-fold).

### 2.3. Validation of MQTL by GWAS-Based Marker–Trait Associations (MTAs)

**Figure 4:**
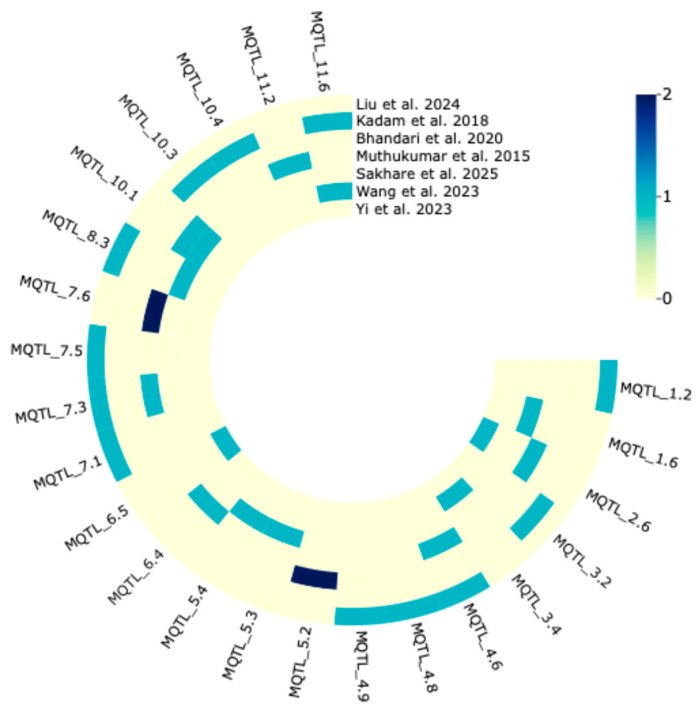
Validation of MQTL by MTA numbers on rice drought-related traits from GWAS with seven diverse natural populations. Color codes showed the number of MTAs overlapping the identified MQTL [72,73,74,75,76,77,78].

To validate the reliability of the identified meta-QTLs, we assessed their co-localization with significant single-nucleotide polymorphisms (SNPs) from an independent genome-wide association study (GWAS) (Figure 4). The physical intervals of the 77 MQTLs were defined as the peak position ± 0.5 Mbp. These intervals were screened against a compiled dataset of marker–trait associations (MTAs) from seven drought-tolerant GWAS (2015–2025), which encompassed 1128 rice accessions across seven independent panels (120–305 per panel). An MQTL was considered validated if it overlapped with at least one significant SNP (−log10(*p*) ≥ 5.0, resulting in the confirmation of 23 MQTLs (29.8%) by MTAs. Each validated MQTL co-localized with one to two MTAs.

### 2.4. Comparative Analysis of Drought Tolerance, Root Architecture, and Physiological Responses Between Wild-Type and T608 Mutant Rice

**Figure 5:**
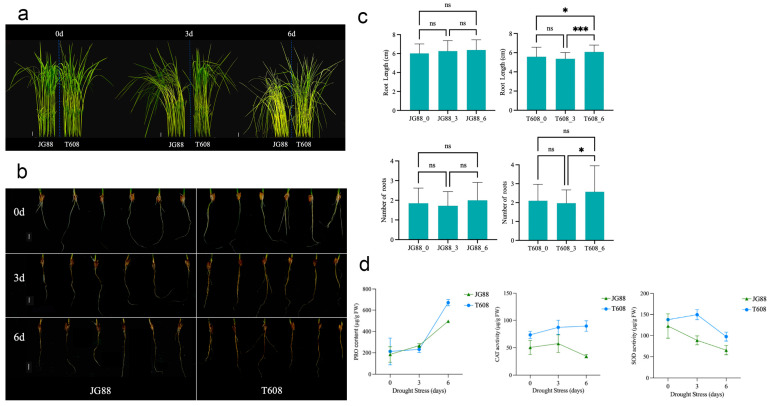
Comparative analysis of drought tolerance between wild-type (JG88) and mutant (T608) rice at the seedling stage. (**a**) Shoot and (**b**) root phenotypes of plants at 0, 3, and 6 days after drought treatment. Scale bar = 1 cm. (**c**) Dynamic changes in root length and root number of wild-type (JG88) and mutant (T608) plants. Data are presented as mean ± SD (n ≥ 3). * *p* < 0.05, *** *p* < 0.001 (Student’s *t*-test). (**d**) PRO content and CAT and SOD activities in roots of JG88 and T608 at 0, 3, and 6 days of drought stress.

The T608 mutant exhibited pronounced morphological advantages under drought stress. At the seedling stage, following simulation of drought with polyethylene glycol 6000 (PEG 6000), a clear phenotypic divergence was observed. After 3 days, JG88 (wild-type) leaves showed mild wilting, characterized by curled tips and drooping, whereas T608 leaves remained largely upright with only slight symptoms. By day 6, JG88 plants were severely wilted, in contrast to T608, which maintained a markedly superior overall condition despite some leaf curling (Figure 5a). Root morphology was examined 6 days after treatment (Figure 5b); scale bar = 1 cm, consistent across samples. T608 developed a root system with significantly greater length and number than JG88, which showed no significant changes (*p* > 0.05), indicating enhanced root development in T608 under drought stress (Figure 5c).

The physiological basis of the enhanced drought tolerance was further investigated by profiling the proline (PRO) content and the activities of the antioxidant enzymes catalase (CAT) and superoxide dismutase (SOD) at 0, 3 and 6 days. PRO accumulation was significantly higher in T608 than in the wild-type JG88 by day 6. CAT activity was elevated in T608 at days 0 and 3 and remained high at day 6, in contrast to a sharp decline observed in JG88. SOD activity, in contrast, was induced in T608 at day 3 and subsequently declined at a slower rate, resulting in a significantly higher final level than in JG88 at day 6 (Figure 5d). These findings demonstrate that the drought resilience of T608 is orchestrated not only at the morphological level but also through a physiological capacity, involving enhanced osmotic adjustment and a more potent antioxidant defense system.

### 2.5. RNA-Seq, Functional Enrichment (GO and KEGG) of DEGs and qRT-PCR Validation

**Figure 6:**
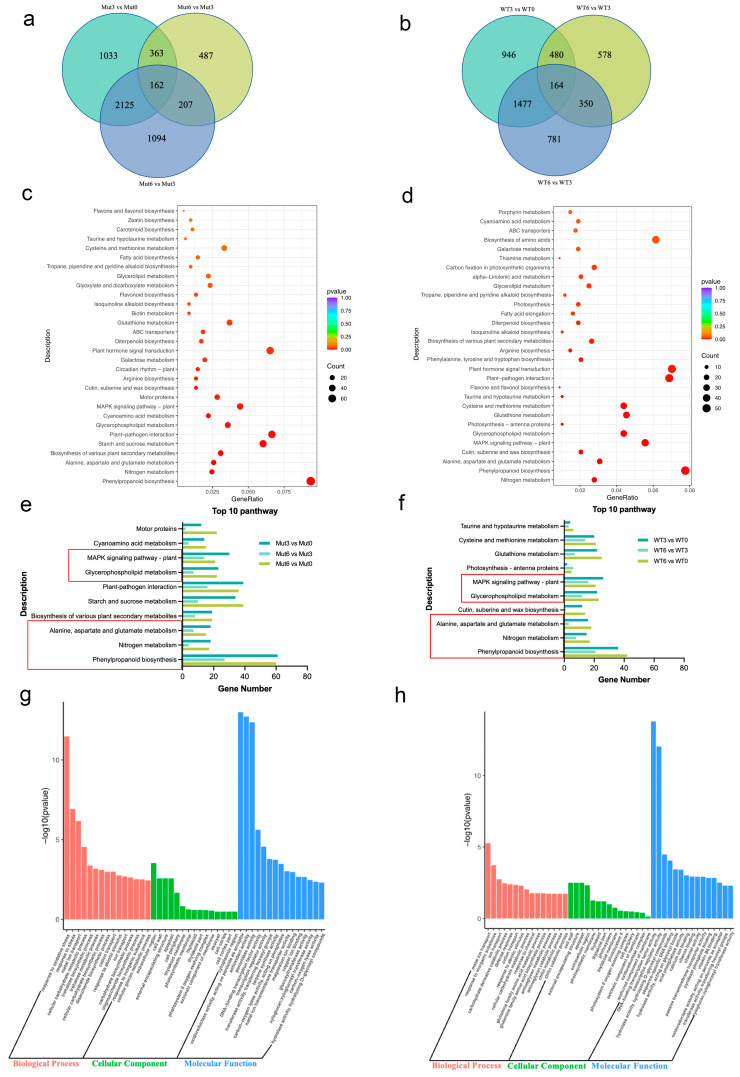
Transcriptomic profiling and functional enrichment of differentially expressed genes (DEGs) in JG88 and T608 under drought stress. (**a**,**b**) Venn diagrams illustrating the distribution of DEGs in (**a**) T608 and (**b**) JG88 across three time points. (**c**,**d**) KEGG pathway enrichment analysis of DEGs in (**c**) T608 and (**d**) JG88. (**e**,**f**) The top 10 significantly enriched KEGG pathways for (**e**) T608 and (**f**) JG88. Red boxes highlight pathways common to both genotypes. (**g**,**h**) Gene Ontology (GO) term enrichment analysis for (**g**) T608 and (**h**) JG88.

To elucidate the molecular mechanisms underlying the observed drought tolerance, transcriptome analysis was performed on roots of T608 and JG88 under drought stress at 0, 3 and 6 d. Comparative analysis identified distinct transcriptional landscapes between the mutant and wild-type, as visualized by Venn diagrams of differentially expressed genes (DEGs) at each time point (Figure 6a,b). KEGG pathway enrichment analysis revealed that the top 10 significantly enriched pathways differed markedly between T608 and JG88 (Figure 6c,d). The mutant-unique pathways included biosynthesis of various plant secondary metabolites, starch and sucrose metabolism, motor proteins, cyanoamino acid metabolism, and plant–pathogen interaction (Figure 6e,f), all of which have established roles in drought adaptation. GO term analysis further corroborated this, identifying mutant-specific enrichments in response to oxidative stress, response to stress, extracellular region, oxidoreductase activity, acting on peroxide as acceptor, antioxidant activity, and peroxidase activity (Figure 6g,h).

**Figure 7:**
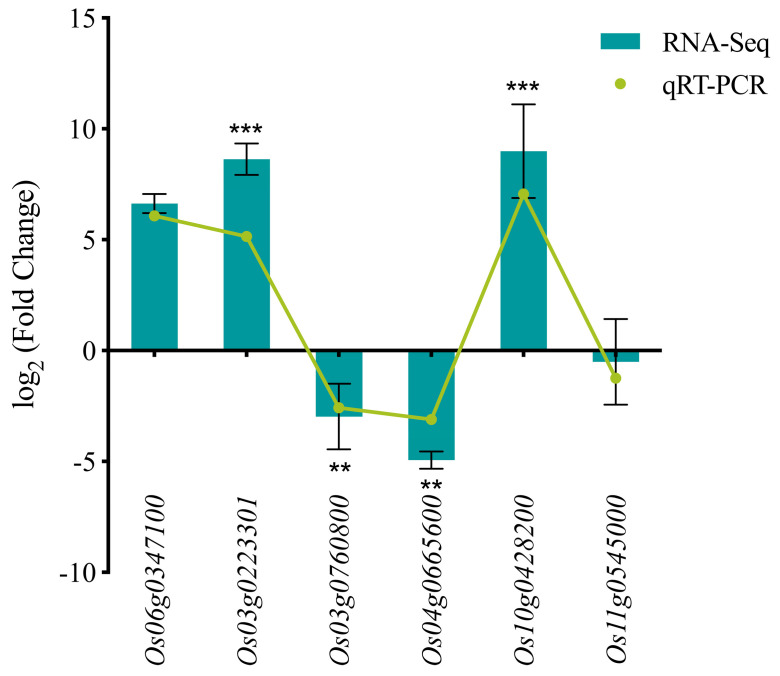
Validation of RNA-Seq expression profiles by qRT-PCR. Data are presented as mean ± SD (*n* = 3). Significance levels are denoted as ** *p* < 0.01, *** *p* < 0.001.

The reliability of the transcriptome data was independently validated by quantitative reverse transcription PCR (qRT-PCR) analysis of six randomly selected genes. The expression patterns determined by qRT-PCR were consistent with the RNA-seq results (Figure 7). The transcript abundance of *Os06g0347100*, *Os03g0223301* and *Os10g0428200* was significantly up-regulated, while that of *Os03g0760800*, *Os04g0665600* and *Os11g0545000* was significantly down-regulated. The strong concordance in gene expression trends, despite minor differences in absolute values between platforms, confirms the robustness of the transcriptome data.

### 2.6. Integrative RNA-Seq, MQTL, and Clustering Analyses Reveal Mutant-Specific Expression Modules

**Figure 8:**
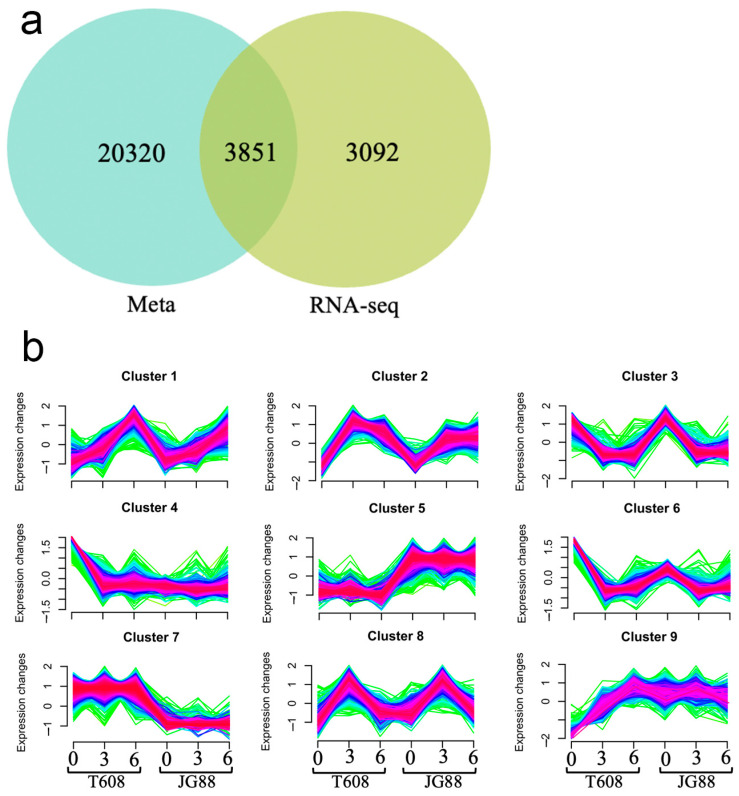
Integrated analysis of transcriptomic data and meta-QTLs identifies a mutant-specific drought-response module. (**a**) Venn diagram illustrating the intersection between drought-responsive differentially expressed genes (DEGs) from RNA-seq and genes located within meta-QTL (MQTL) regions. (**b**) Temporal expression patterns of the 3851 intersecting genes, resolved into nine clusters by c-means fuzzy clustering analysis.

An integrated analysis was performed to identify high-confidence candidate genes by combining transcriptomic data with the stable genomic regions defined by MQTLs. The initial drought-responsive DEG set was derived from two key comparisons: T608 versus JG88 across all time points (2821 DEGs) and T608 across different time points (5471 DEGs) (Figure S1). The union of these DEGs was then intersected with genes located within the MQTL intervals, yielding a refined set of 3851 genes that are both differentially expressed and co-localized with stable genetic loci (Figure 8a).

**Figure A1:**
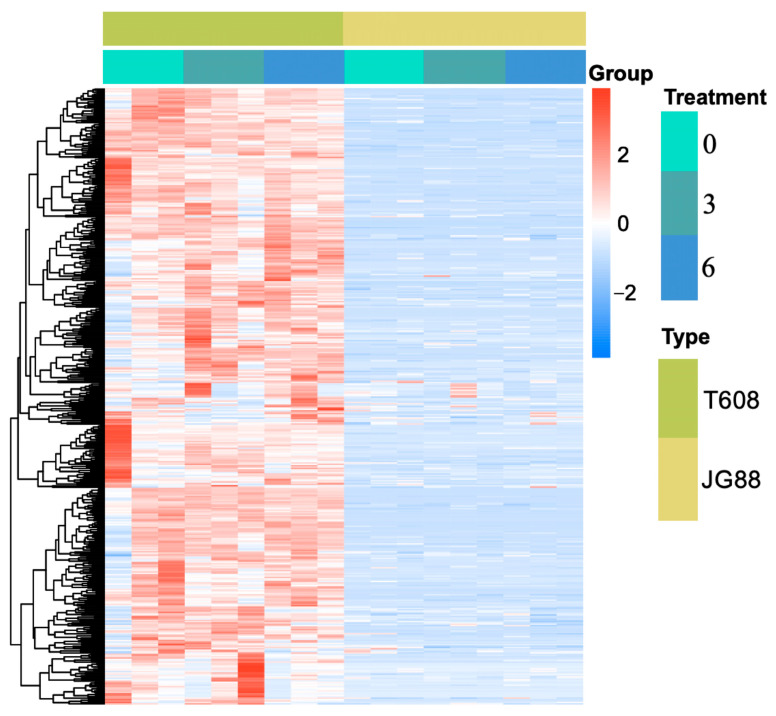
Integrated transcriptomic and meta-QTL analysis uncovered a mutant-specific drought-response module, illustrated by a heatmap of Cluster 7 expression profiles in JG88 and T608 under 0, 3, and 6 days of drought stress (*n* = 3 biological replicates).

The expression patterns of these 3851 genes were analyzed by c-means fuzzy clustering, which resolved nine temporally distinct expression clusters (Figure 8b). Cluster 7 was particularly noteworthy, as it comprised genes with transcript levels that increased in the T608 but decreased in the JG88 under drought stress, representing a clear mutant-specific, drought-induced expression signature. The accompanying dendrogram confirmed tight clustering of biological replicates, and the corresponding heatmap demonstrated a pronounced up-regulation (red shift) in the mutant samples, corroborating the distinct transcriptional response of this genotype (Figure A1).

### 2.7. Screening of Drought-Responsive Differentially Expressed Candidate Genes (DECGs) in Rice Based on Cis-Acting Regulatory Elements (CREs)

The promoter sequences of the DEGs in Cluster 7, which were obtained through c-means fuzzy clustering, were analyzed for cis-acting regulatory elements (CREs) (Figure S2). Functional screening against drought-related annotations led to the identification of nine significantly enriched CREs. The enriched CREs included ABRE (ABA-responsive element), DRE core (dehydration-responsive element core sequence), MBS/MYB/MYC (MYB-binding site), ARE (anaerobic-responsive element), W box (WRKY-binding site), as-1 (activation sequence-1), and the G-box (CACGTG core motif). A high-confidence subset of candidate drought-responsive differentially expressed candidate genes (DECGs) was obtained by screening for the ABRE cis-acting element within Cluster 7, and this gene set served as the basis for subsequent functional analysis.

### 2.8. Hub Proteins for Drought Tolerance Identified via PPI Network and AlphaFold Structure Analyses

**Figure 9:**
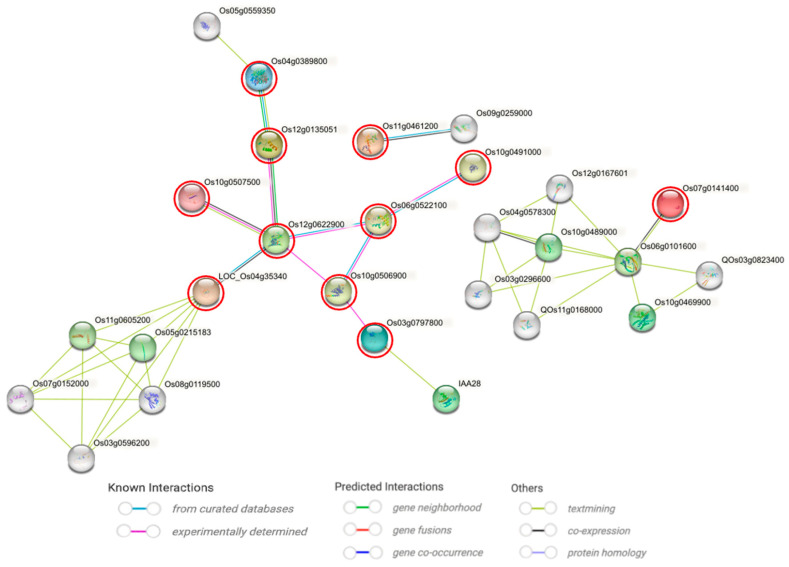
Protein–protein interaction (PPI) network of drought-responsive differentially expressed candidate genes (DECGs). The network was constructed using the STRING database (minimum interaction score: 0.150), depicting potential functional associations among the encoded proteins. Hub proteins, representing central nodes with the highest connectivity, are highlighted. Colored nodes represent the query proteins (red circle, i.e., the target proteins in this study) and first-shell interactors, whereas white nodes denote second-shell interactors.

A protein–protein interaction (PPI) network was constructed using the proteins encoded by the DECGs. Network analysis identified eleven hub proteins (Os04g0389800, Os12g0135051, Os12g0622900, Os10g0507500, Os07g0141400, Os06g0522100, Os10g0491000, Os10g0506900, Os03g0797800, LOC_Os04g35340, and Os11g0461200). Notably, LOC_Os04g35340 and Os07g0141400 were found to interact with previously reported proteins involved in combined abiotic stress and photosynthesis, respectively. It is important to note that these hub protein candidates require further functional validation. (Figure 9).

**Figure 10:**
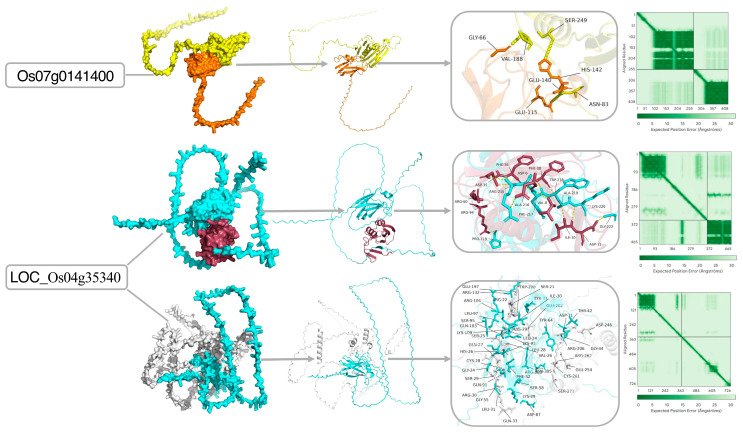
Structural analysis of hub proteins using AlphaFold predictions. Three-dimensional structures were predicted by AlphaFold and visualized in PyMOL. For each protein, the analysis integrates multiple representations: surface model, cartoon model, key residue interactions, and predicted alignment error (PAE) plot.

To gain structural insights into their molecular functions, the three-dimensional structures of two hub proteins were modeled using AlphaFold and visualized by PyMOL (Figure 10). Surface and cartoon representations provided an intuitive overview of the proteins’ spatial architecture, facilitating analysis of their structural foundations for potential interactions. Examination of the three-dimensional conformations and key residue annotations enabled rapid localization of putative PPI interfaces. The reliability of these structural models was further assessed using AlphaFold’s predicted alignment error (PAE) plots, which estimate the confidence in the relative spatial positioning of residue pairs. Analysis of the PAE plots revealed that Os07g0141400 exhibited uniformly low PAE values across the full length, indicating a high-confidence global fold compatible with a stable single-domain architecture. In contrast, LOC_Os04g35340 displayed low PAE values exclusively within a well-defined diagonal block, corroborating the exceptional accuracy of the predicted core domain, whereas the flanking regions were associated with moderately higher uncertainty. Thus, the variations in structural confidence revealed by the PAE analysis provide a structural basis for hypothesizing functional differentiation among these hub proteins.

## 3. Discussion

### 3.1. Identification of QTLs Associated with Drought Tolerance and Construction of MQTLs in Rice

The sustainability of rice production in China is increasingly threatened by a fundamental contradiction: the crop’s high water demand is starkly at odds with growing water scarcity, rendering traditional flooded cultivation unsustainable [79]. Developing water-saving and drought-resistant rice varieties is therefore paramount to ensuring food security.

Conventional QTL mapping, however, is often plagued by unstable genetic effects and wide CIs across different populations and environments [80]. To overcome these limitations, meta-QTL (MQTL) analysis has emerged as a powerful approach that integrates disparate QTL studies to distill stable genomic regions with refined intervals and enhanced reliability [81]. The efficacy of this strategy is well-documented, having been successfully applied to refine genetic maps for complex traits in various crops, including foxtail millet, rice, and others [12,82].

This study establishes a high-resolution genetic landscape for drought tolerance in rice by integrating 901 initial QTLs from 52 independent studies into 77 stable MQTLs. Furthermore, our analysis covered a broader spectrum of 15 traits, with root architecture—the primary organ for water and nutrient acquisition—emerging as the most heavily represented trait category, underscoring its pivotal role in drought adaptation [83]. Here, the most significant refinement achieved was the dramatic compression of CIs. The MQTLs exhibited CIs that were 2.44- to 20.4-fold narrower than the original QTLs, with an average reduction of 16.7 cM, resulting in a final average CI of 3.63 cM. This aligns with the established consensus that CI narrowing is a universal strategy for enhancing the mapping accuracy of complex traits, and this point is consistently supported by rice research [84,85]. Consequently, our study achieves a dual breakthrough in both the scale of integration and the precision of localization, providing a robust genetic framework for the fine-mapping of drought tolerance genes in rice.

On this basis, we benchmarked our dataset against four major MQTL studies released in recent years. Compared to Nurhanis Selamat et al. [16], we included 32 additional articles, bringing the total number of initial QTLs from 512 to 901 and the number of MQTLs from 70 to 77. This expanded dataset also encompasses new trait categories, including spikelet/panicle architecture, bioregulation, tillering, and germination. In comparison with B. P. Mallikarjuna Swamy et al. [12] (seven overlapping articles), we raised the grain-yield QTL count from 53 to 76. In their study, they only found five MQTLs that could apply Marker-Assisted Selection (MAS) and a pyramiding program in rice. Compared to the work of Bahman Khahani et al. [86], our study identified 338 additional QTLs and 16 additional MQTLs. The mean confidence interval (CI) was reduced by 1.85 cM (from 5.48 cM to 3.63 cM). In comparison to Parisa Daryani et al. [87], our integrated dataset of 52 studies included only 26 that overlapped with those in their study, with the remainder being distinct. Consequently, the MQTLs we identified not only exhibited smaller average confidence intervals on ten chromosomes but also showed significant differences in their physical genomic locations. Collectively, our study advances the field across four axes—literature coverage, QTL/MQTL number, CI compression, and trait breadth—providing a narrower and more robust genetic trait basis for fine-mapping and cloning drought tolerance genes in rice.

### 3.2. The Drought-Tolerant Mutant t608 Exhibits Enhanced Root Architecture and Antioxidant Capacity

The elite *japonica* rice variety JG88 is prized for its high yield and grain quality and has been the subject of studies aimed at improving its disease resistance and drought tolerance [88,89]. Despite these efforts, it remains deficient in robust stress resistance resources, and its improvement via conventional breeding is challenging. To address this, we generated a mutant population from JG88 using ginseng DNA, from which the line T608 emerged with pronounced drought tolerance. This study compared the physiological and molecular responses of T608 and its wild-type progenitor, JG88, under drought stress simulated by polyethylene glycol (PEG-6000). The method is well-established for simulating soil water deficit in plant drought tolerance research [90,91].

Under PEG-simulated drought stress, the T608 mutant showed significantly improved root morphological traits compared to JG88, as measured by root length and number. This increase in root length and number is critically important, as the root system acts as the primary sensor of soil water deficit, and its adaptive plasticity directly constrains canopy performance, thereby determining yield under drought stress [92,93,94].

At the physiological level, T608 demonstrated a superior capacity to mitigate oxidative damage. We observed that the mutant sustained higher activities of key antioxidant enzymes, including superoxide dismutase (SOD) and catalase (CAT), along with greater accumulation of the osmoprotectant proline. This enhanced antioxidant defense is crucial for scavenging drought-induced reactive oxygen species (ROS), thereby maintaining cellular homeostasis and protecting membrane integrity [95,96,97,98]. Our findings align with the established consensus that drought-tolerant genotypes maintain high protective enzyme activity and osmolyte accumulation to alleviate oxidative membrane damage [99]. The concerted enhancement in both root foraging capacity and cellular detoxification capability provides a compelling physiological explanation for the observed drought tolerance of the T608 mutant.

### 3.3. Expression Signatures and Physiological Traits Jointly Suggest Potential Metabolic Adjustments in T608 Under Drought Stress

Our transcriptomic analysis revealed that the drought tolerance of the T608 mutant is underpinned by a coordinated up-regulation of key metabolic pathways. The induction of the phenylpropanoid pathway could enhance the synthesis of flavonoids, which may complement the observed increases in CAT and SOD activities by providing non-enzymatic antioxidants to mitigate oxidative damage [100,101]. Furthermore, the activation of cyanogenic amino acid metabolism provides a plausible source for the accumulated proline, contributing to both osmotic adjustment and non-enzymatic ROS scavenging [102,103]. Beyond immediate osmotic adjustment, the drought-induced up-regulation of ADP–glucose pyrophosphorylase (AGPase) channels assimilates into starch, thereby expanding early-grain filling reserves and potentially buffering yield under sustained water deficit [104,105].

Taken together, our data support a model where T608’s drought tolerance may stem from a synergistic ROS-scavenging system. This system appears to integrate enzymatic functions (e.g., CAT, SOD) with non-enzymatic antioxidant activities (e.g., potential flavonoids and proline) [101,106], with the extracellular region being a key site for this coordinated defense [107,108]. In summary, the convergence of enhanced antioxidant capacity, proline-mediated osmoprotection, and resilient carbon metabolism constitutes the core metabolic strategy that bolsters drought tolerance in the T608 mutant.

### 3.4. Integrated Analyses Identify Drought-Hub Genes with Putative Roles in Drought Response

The integration of meta-QTL mapping with transcriptomic dynamics provided a powerful filter to distill a vast number of drought-responsive genes into a manageable set of high-confidence candidates. Our integrated bioinformatics analyses—through sequential application of existing tools for co-expression clustering (Mfuzz), cis-element profiling, PPI network analysis, and structural assessment—progressively refined the candidate list from 3851 MQTL-localized DEGs to just eleven hub genes whose protein products form the central nodes of the interactome (Figure 10). This systematic approach effectively prioritizes genes that are not only genetically associated with drought tolerance across diverse backgrounds but also exhibit dynamic transcriptional regulation and occupy central positions in stress-responsive protein networks.

The enrichment of drought-related cis-elements (including key elements such as ABRE and DRE core; see Figure S2 for details). This cis-regulatory signature formed the basis for a stepwise screening approach, which identified eleven high-confidence drought-responsive hub genes. These included *Os04g0389800* (encoding a probable acetolactate synthase 2), *Os12g0135051* (pentatricopeptide repeat-containing protein), Os12g0622900 (Mov34/MPN/PAD-1 family protein), *LOC_Os04g35340* (MrBTB1, a BTB-MATH domain protein), *Os06g0522100* (plant peroxidase domain-containing protein), *Os10g0491000* (plant basic secretory protein), *Os03g0797800* (auxin-responsive protein IAA14), *Os07g0141400* (23 kDa polypeptide of photosystem II), and *Os11g0461200* (UDP-glucosyltransferase domain-containing protein). Notably, *Os10g0506900* and *Os10g0507500* are annotated only as “expressed proteins” with unknown function. Based on functional annotation and interaction profiles, we prioritized *LOC_Os04g35340* and *Os07g0141400* as candidate genes (CGs). PPI analysis revealed that LOC_Os04g35340 exhibits significant associations with previously reported genes involved in combined abiotic stress responses [109]; Os07g0141400 displays robust interactions and pronounced co-expression with photosynthesis-related genes that are specifically activated upon *Trichoderma harzianum* treatment [110]. These two hub proteins serve as central nodes in regulatory networks, as evidenced by the presence of drought-related cis-elements in their promoters and their interactions with established stress-associated proteins.

To explore the structural basis of the observed interactions, we integrated AlphaFold-predicted structures with network analysis to formulate initial, structure-based hypotheses for the observed interactions. Although predictive, these models generate testable residue-level hypotheses that provide a concrete foundation for future functional studies, such as site-directed mutagenesis to validate critical interaction residues [111,112].

## 4. Materials and Methods

### 4.1. Data Collection and Screening of Drought-Related QTLs

Information on QTLs underlying rice drought tolerance and their flanking molecular markers was assembled from peer-reviewed publications. A total of 52 reports published up to 2023 that performed QTL mapping for drought-related traits were retained, encompassing 901 distinct QTLs. These QTLs cover a wide spectrum of characteristics—including root, spikelet/panicles, grain yield, leaves, flowers, shoots, bioregulation, tiller, and biomass—that respond to altered plant water potential. Only QTLs detected specifically under drought-stress treatments were extracted; loci identified under well-watered controls were omitted as irrelevant to the present objective. After curation, the parents used in the populations, the type of populations, the chromosomal position, LOD score, PVE, and flanking markers (SSR, SNP or InDel) of each QTL were integrated into a single dataset and projected onto a consensus map using BioMercator v4.2 for comparative visualization and downstream meta-analysis.

### 4.2. Integration of Rice Reference Genetic Maps and Construction of a Consensus Map for the Localization of Drought-Tolerance-Related QTLs

To integrate rice genetic linkage maps and achieve precise localization of quantitative trait loci (QTLs), this study selected 10 rice accessions from the Gramene database (https://archive.gramene.org, accessed on 3 September 2024) that encompassed the following SSR markers: CIAT SSR2006, Cornell SSR 2001, Hokka 2000, IGCN1998, IRMI2003, JRGPRFLP2000, KRGRP1998, Niigata2000, TTU CTIR2000, and TTU IRIR2000. To ensure consistency in data format, all genetic map data were converted to the standard GenBank Feature Format (GFF). During the map integration phase, the LPmerge software (version 1.7) was used. This software, based on linear programming algorithms, efficiently merges multiple genetic maps while maintaining consistency in marker order. Subsequently, the BioMercator V4.2.3 software was used to perform an in-depth integration of the preliminarily integrated map and an additional 18 genetic maps, ultimately constructing a consensus map. Based on the constructed consensus map, QTLs were accurately projected onto the map using key metrics, including LOD scores, PVE, confidence intervals, and positional information. The 95% confidence intervals for each QTL were calculated [113].

$$\begin{array}{l} CI=530/\left(N\times R^{2}\right)\\ CI=163/\left(N\times R^{2}\right)\\ CI=97.462/{\left(N\times R^{2}\right)}^{0.835} \end{array}$$

### 4.3. Meta-QTL Analysis

MQTL analysis was performed using Biomercator v4.2 software. Two algorithms were selected based on the number of QTLs on each chromosome: the Goffinet and Gerber method was adopted when the number of QTLs was ≤10, while the Veyrieras method was used when the number exceeded 10 [114,115]. This analysis determined the number of potential MQTLs on each chromosome via five criteria: AIC, AICc, AIC3, BIC, and AWE. A model was identified as the optimal one if it achieved the minimum value in at least three of these five criteria, and based on this, the 95% confidence interval and peak position of MQTL were defined. For QTL integration, the peak of the original QTL was required to lie within the confidence interval of the corresponding MQTL; a QTL was classified into a specific MQTL if its membership probability to that MQTL exceeded 60% [116]. Subsequently, the “QTL-overview index” method was employed to estimate the probability of QTL occurrence in each segment of the reference map, with a unit of 0.5 cM [117].

### 4.4. Comparative Analysis of MQTLs with Results from Drought Tolerance-Associated Genome-Wide Association Studies (GWAS)

All MQTLs identified were mapped to the rice reference genome. Initially, flanking markers of each MQTL were manually retrieved, and their primer sequences were obtained from the Gramene database (https://archive.gramene.org/, accessed on 1 December 2024). Subsequently, these flanking markers and their primer sequences were subjected to BLAST alignment (version 2.12.0+) with the rice reference genome sequences in the RAP-DB database (https://rapdb.dna.affrc.go.jp/, accessed on 2 December 2024) to acquire the physical coordinates of the markers. For markers without annotated physical coordinates, manual anchoring was adopted to determine their physical positions. Finally, a review of drought tolerance-associated GWAS studies (Liu et al. 2024 [72], Kadam et al. 2018 [73], Bhandari et al. 2020 [74], Muthukumar et al. 2015 [75], Sakhare et al. 2025 [76], Wang et al. 2023 [77], Yi et al. 2023 [78]) was conducted to collect the reported SNP peak loci for analysis of their overlap with the identified MQTLs (Table S1).

### 4.5. Plant Materials and Drought Treatments

Seeds of JG88 (wild-type) and T608 (mutant) were provided by Yanbian University, Jilin, China. The T608 mutant was developed by intergeneric hybridization between rice (*Oryza sativa* L. cv. JG88) and ginseng (*Panax ginseng*). Briefly, ginseng genomic DNA was introduced into emasculated JG88 florets using the pollen-mentor effect; immature hybrid embryos were rescued in vitro to obtain F_1_ plants. The progeny were advanced through six successive generations of self-pollination under field conditions, resulting in the genetically stable mutant line T608. Rice seeds were treated with 3% H_2_O_2_ to break dormancy and then kept in the dark for 24 h before being transferred to a biochemical incubator. They were germinated in the dark at 28 °C for 48 h. The germinated seeds were transplanted into 96-well hydroponic trays (96 plants per tray) and cultivated using Yoshida rice nutrient solution in a plant incubator. The experiment consisted of 2 genotypes (wild-type JG88 and the t608 mutant line) and 3 sampling time points (0, 3, and 6 days after PEG addition), with 3 biological replicates at each time point, totaling 18 trays (1728 plants). The seedling cultivation conditions included a photoperiod of 13/11 h (light/dark cycle), a light intensity of 26,000 Lux, a humidity level of 75%, and a temperature cycle of 28/25 °C. When the seedlings reached the three-leaf stage, drought stress was simulated using 20% polyethylene glycol 6000 (PEG-6000). Root samples were randomly collected from each biological replicate; root length and root number were measured using 70 independent roots per sample. The samples were rapidly frozen in liquid nitrogen and stored at −80 °C for subsequent RNA transcriptomic analysis. All physiological assays were performed with three biological replicates (*n* = 3). Significance was determined by two-tailed Student’s *t*-test using GraphPad Prism v10.0; *p* < 0.05 was considered significant.

### 4.6. RNA Sequencing

RNA-seq library preparation and sequencing were performed by Novogene Co., Ltd. (Beijing, China). DEGs were identified using thresholds of |log_2_FC| ≥ 1 and FDR < 0.05. Transcript or gene expression levels were quantified in terms of FPKM (Fragments Per Kilobase of exon model per Million mapped fragments). The calculation formula is as follows: $FPKM=10^{6}C/NL/10^{3}$, where C denotes the number of sequenced fragments; N denotes the number of sequenced fragments aligned to reference genes; and L denotes the number of base pairs in the gene. Gene functional annotation of the assembled gene sequences was conducted in various databases to elucidate the functions of different genes.

### 4.7. Quantitative Real-Time Polymerase Chain Reaction (qRT-PCR) Validation

To assess the reliability of RNA sequencing data and validate the expression patterns of DEGs, six genes with differential expression patterns were randomly selected and validated by quantitative reverse transcription polymerase chain reaction (qRT-PCR) analysis. Primers were designed based on the corresponding sequences in the RAP-DB database (https://rapdb.dna.affrc.go.jp/) using Primer Premier 5.1 software; primer sequences are listed in Table S2. Total RNA was reverse-transcribed into cDNA using the StarScript II First-Strand cDNA Synthesis Kit-II (GeneStar, Beijing, China). Quantitative validation was performed using the TB Green^®^ Premix Ex Taq™ (Tli RNaseH Plus) kit (Takara Biomedical Technology (Beijing) Co., Ltd., Beijing, China), with cDNA as the template and *ACT11* and *UBC* as internal reference genes, in three independent replicates per sample. The relative expression level, expressed as fold change (FC), was calculated using the $2^{-∆∆CT}$ method for qRT-PCR data and as $2^{{log}_{2}FoldChange}$ for RNA-seq data (analyzed with the DESeq2 R package, version 1.20.0). An FC > 1 indicates up-regulation, whereas an FC < 1 indicates down-regulation. All data analysis and graphical presentations were performed using GraphPad Prism 10.0 software.

### 4.8. Screening of Differentially Expressed Candidate Genes (DECGs) Based on Cis-Acting Element Analysis

Genomic sequences and annotation information for cis-acting elements were retrieved from the Ensembl Plants database (*Oryza sativa Japonica* Group, IRGSP-1.0 version) to ensure sequence accuracy and integrity. The promoter region was defined as follows: the sequence from 2000 bp upstream of the start codon (ATG) in the gene coding region to the transcription start site (TSS); if the length of the 5′ untranslated region (5′UTR) of some genes exceeded 100 bp, the promoter region was adjusted to the sequence from 1500 bp upstream of ATG to the start site of 5′UTR to avoid interfering with the cis-element prediction analysis. Using the “Sequence Extraction” function of TBtools (Version 1.120), the rice genome sequences in FASTA format were imported into the software. After matching the chromosomal positions and coordinates of candidate genes via the “Gene ID Mapping” function, batch extraction of promoter sequences was performed according to the aforementioned criteria. Ten randomly selected sequences were verified through NCBI BLAST, showing ≥99% consistency with the reference genome (IRGSP-1.0), which ruled out errors caused by sequence truncation or annotation deviations.

### 4.9. Systematic Screening of Hub Proteins via PPI Network Construction and Visualization of AlphaFold-Predicted Tertiary Structures

Protein IDs of 24 candidate proteins encoded by DECGs were imported into the STRING database, with the species restricted to *Oryza sativa* (rice). A high-confidence interaction score threshold (≥0.7) was set to identify interactions supported by experimental evidence, co-expression, and homology prediction.

Tertiary structures were predicted using the AlphaFold 3 open-source model. FASTA-formatted sequences of the candidate proteins were input, and parameters were set as model_type = 5, num_recycles = 3, and use_templates = True to enhance prediction accuracy. PyMOL software (Version 2.5.2) was utilized for structural visualization: the spatial locations of conserved functional domains were labeled, and the spatial arrangement of key amino acid residues was analyzed.

## 5. Conclusions

In this study, by meta-analyzing 901 drought-related QTLs from 52 independent studies, we condensed them into 77 meta-QTLs with an average confidence interval of 3.63 cM—an 82% reduction compared with the original QTLs. Twenty-three of these MQTLs co-localized with GWAS-significant SNPs, reinforcing their robustness across diverse germplasm. Integration with RNA-seq data further identified 3851 differentially expressed genes residing within the MQTL intervals. A stepwise approach narrowed this to eleven high-confidence hub genes. Based on functional annotation and interaction profiles, we prioritized *LOC_Os04g35340* and *Os07g0141400* as CGs. PPI analysis revealed that LOC_Os04g35340 exhibits significant associations with previously reported genes involved in combined abiotic stress responses; Os07g0141400 displays robust interactions and pronounced co-expression with photosynthesis-related genes that are specifically activated upon *Trichoderma harzianum* treatment. These CGs represent high-potential targets for future functional validation and could inform the development of molecular markers, ultimately accelerating the breeding of drought-resilient rice varieties.

## Figures and Tables

**Table 1:** Summary of QTL studies associated with drought tolerance in rice used for meta-QTL analysis.

Parents	Population Type ^a^	Population Size	No. of Markers	Marker Type ^b^	References
IR77298-5-6-18/2*Sabitri	BC_1_	294	124	SSR	(Yadaw et al. 2013) [19]
Xiaobaijingzi/Kongyu 131	F_2:7_ RILs	220	104	SSR	(Xing, Zhao, and Zou 2014) [20]
Samgang/Nagdong	DH	101	185	SSR, STS	(Kim et al. 2017) [21]
IR64 X APO	BILs	50	25	SSR	(Baghyalakshmi et al. 2016) [22]
Kali Aus/2*IR64,Kali Aus/2*MTU1010	BC_1_F_4_	300	600	SSR	(Sandhu et al. 2014) [23]
IR55419-04/2*TDK1	BC_1_F_3:4_	365	600	SSR	(Dixit et al. 2014) [24]
Miyang 23/Jileng 1	RIL	253	291	SSR	(Chen et al. 2023) [25]
CT9993-5-10-1-M/IR62266-42-6-2	DH	154	280	RELP, AFLP, SSR	(Songping et al. 2011) [26]
CR 143-2-2/Krishnahamsa	RILs	190	21	SSR	(Barik et al., n.d.) [27]
Gharib/Sepidroud	F_2:4_	148	575	SSR	(Zahra Mardani-2013) [28]
Zheshan97B/IRAT109	F_10_	187	213	SSRs	(G.L. Liu-2008) [29]
Banglami/Ranjit	F_4_	90	94	SSR	(Vinay Sharma-2017) [30]
IR64/Khazar	BC_2_F_2_	208	83	SSR	(CHEN Man-yuan-2011) [31]
Zhenshan 97B/IRAT109	RILs	195	213	SSR	(HU Song-ping-2007) [32]
IR 58821/IR 52561	RILs	148	399	RFLP, AFLP	(A. Manickavelu-2006) [33]
Swarna/WAB 450-I-B-P-157-2-1	BIL	202	412	SSR	(Saikumar et al. 2014) [34]
CR143-2-2/Krishnahamsa	RIL	190	201	SSR	(Barik et al. 2018) [35]
Apo/Moroberekan	BC_1_F_3_	289	108	SSR, STS	(Reena Sellamuthu-2015) [36]
CT9993-510-1-M/IR62266-42-6-2	DH	154	315	RELP, AFLP, SSR	(Nguyen et al. 2004) [37]
Zheshan97B/IRAT109	RILs	187	213	SSR	(Liu et al. 2010) [38]
IR58821–23-B-1–2-1/IR52561-UBN-1–1-2	RIL	166	399	AFLP, RFLP	(Ali et al. 2000) [39]
Vandana/Way Rarem	F_3_	436	126	SSR	(Bernier et al. 2007) [40]
IAC 165/CO39	RIL	125	182	RFLP, SSR	(Courtois et al. 2003) [41]
Shennong265/Haogelao	RIL	94	130	SSR	(Gu et al. 2012) [42]
IR64/Azucena	DH	56	175	RFLP	(Hemamalini, Shashidhar, and Hittalmani 2000) [43]
Akihikari/IRAT109	BILs	106	113	SSR	(Horii et al. 2006) [44]
CT9993/IR62266	RILs	184	399	RFLP, AFLP	(Kamoshita et al. 2002) [45]
CT9993/IR62266	DH	220	315	RELP, AFLP	(Kamoshita et al. 2002) [46]
Akihikari/IRAT109	BILs	106	57	SSR	(Kato et al. 2008) [47]
CT9993/IR62266	DH	154	315	AFLP	(Kumar, Venuprasad, and Atlin 2007) [48]
CT9993/IR62266	DH	154	315	RFLP, AFLP, SSR	(Lanceras et al. 2004) [49]
IRAT109/Yuefu	DH	116	336	RELP, SSR	(Li et al. 2005) [50]
Bala/Azucena	RILs	205	1151	SSR	(MacMillan et al. 2006) [51]
Nootripathu/IR20	RIL	250	79	SSR	(Michael Gomez et al. 2010) [52]
KaliAus X IR64 KaliAus X MTU1010	BC	300	600	SSR	(Palanog et al. 2014) [53]
Bala/Azucena	RILs	205	135	RELP, AFLP	(Price et al. 2000) [54]
Bala/Azucena	RILs	140	6	SSR	(Price et al. 2002) [55]
Nootripathu/IR20	RIL	397	79	SSR	(Prince et al. 2015) [56]
Labelle/Black Gora	F2	204	117	RELP	(Redofia and Mackill, n.d.) [57]
IR55419-04/Super Basmati	F2	418	73	SSR	(Sabar et al. 2019) [58]
HKR47/MAS26,MASARB25/Pusa Basmati	F_2:3_	1460	300	SSR	(Sandhu et al. 2013) [59]
IR64/Azucena	BC_3_F_2_	29	60	SSR	(Shen et al. 2001) [60]
Vandana/Cocodrie	F2:3	187	213	InDels, SNP, SSR	(Solis et al. 2018) [61]
CT9993/IR62266	DH	104	315	RFLP, AFLP, SSR	(Tripathy et al. 2000) [62]
Zhenshan97/Minghui63	F2 RIL	240	221	RFLP, SSR	(Xu et al. 2004) [63]
Zhenshan97/IRAT109	RIL	180	245	SSR	(Yue et al. 2006) [64]
IRAT109/Zhenshan97	RIL	154	220	SSR	(You et al. 2006) [65]
IRAT109/Zhenshan97	RIL	180	220	SSR	(Yue et al. 2008) [66]
Zhenshan97/IRAT109	RIL	180	220	SSR	(Yue et al. 2005) [67]
IR1552/Azucena	RILs	150	107	RFLP, AFLP	(Zhang et al. 2001) [68]
R1552/Azucena	RILs	96	103	SSR	(Zheng et al. 2003) [69]
IR64/Azucena	DH	135	135	RFLP	(Zheng et al. 2000) [70]
Azucena/IR64	DH	96	189	RFLP, SSR	(Zheng et al. 2008) [71]

^a^ BC, backcross population; DH, doubled haploid lines; RIL, recombinant inbred lines; F_2_, second filial generation population. ^b^ SSR, single sequence repeats; RFLP, restriction fragment length polymorphism; AFLP, amplified fragment length polymorphism; SNP, single-nucleotide polymorphism; STS, sequence-tagged site.

## Data Availability

The datasets generated and analyzed during the current study are available in the NCBI repository, https://www.ncbi.nlm.nih.gov/sra/PRJNA1335398 (accessed on 28 September 2025), with the accession number PRJNA1335398. The datasets used and analyzed in the current study are available from the corresponding author on reasonable request.

## Supplementary Materials

The following are available online at https://www.mdpi.com/article/10.3390/plants14233645/s1, Figure S1: Analysis of the Distribution of DEGs between Wild-Type (JG88) and Mutant (T608) under Drought Stress; Figure S2: CRE abundance profile in promoters of drought-responsive candidate genes. The heatmap displays the variation in copy number for nine core CRE motifs across the promoters of the differentially expressed candidate genes (DECGs); Table S1: No. of SNPs Overlapping within MQTL; Table S2: Primer sequence of qRT-PCR.

## Abbreviations

The following abbreviations are used in this manuscript:

QTL	Quantitative Trait Loci
MQTL	Meta-QTL
GWAS	Genome-Wide Association Studies
CI	Confidence Interval
DEGs	Differentially Expressed Genes
CREs	Cis-acting Regulatory Elements
PPI	Protein–Protein Interaction
RNA-seq	RNA Sequencing
PVE	Phenotypic Variance Explained
LOD	Logarithm Of the Odds
BC	Backcross Population
DH	Doubled Haploid Lines
RIL	Recombinant Lnbred Lines
F_2_	Second Filial Generation Population
SSR	Single Sequence Repeats
RFLP	Restriction Fragment Length Polymorphism
AFLP	Amplified Fragment Length Polymorphism
SNP	Single-Nucleotide Polymorphism
STS	Sequence-Tagged Site
AIC	Akaike Information Content
AICc	Akaike Information Content Correction
AIC3	Akaike Information Content 3 Candidate Models
BIC	Bayesian Information Criterion
AWE	Average Weight of Evidence
chr	Chromosomes
WT	Wild-Type
JG88	Jigeng 88
PEG 6000	Polyethylene Glycol 6000
PRO	Proline
CAT	Catalase
SOD	Superoxide
qRT-PCR	Quantitative Real-Time Polymerase Chain Reaction
ROS	Reactive Oxygen Species
GO	Gene Ontology
DRE	Drought-Responsive Element
ARE	Anaerobic-Responsive Element
DECGs	Differentially Expressed Candidate Genes
CGs	Candidate Genes
FC	Fold Change

## Appendix A

**Table A1:** Genomic characteristics of the MQTLs identified for drought tolerance in rice.

MQTL	Chr	CI (95%)	No. of QTL	Mean CI (95%) of Initial QTL	Position	Left Marker	Right Marker
MQTL_1.1	1	2.22	11	30.81	125.5	RM8146	RM7466
MQTL_1.2	1	0.69	12	21.43	150.53	RM8103	RM3627
MQTL_1.3	1	1.67	35	25.95	208.15	RM1349	RM246
MQTL_1.4	1	0.13	10	17.57	222.02	RM7650	RM3632
MQTL_1.5	1	3.06	2	4.12	228.15	RM232	RZ730
MQTL_1.6	1	0.1	34	23.38	232.87	RM3440	RM212
MQTL_1.7	1	0.64	36	25.24	264.88	RM3602	RM6292
MQTL_2.1	2	0.41	24	15.08	56.26	RM6069	RM12729
MQTL_2.2	2	1.91	31	31.34	74.48	RM3549	RM3178
MQTL_2.3	2	3.18	9	22.22	125.08	RM3355	RM6617
MQTL_2.4	2	1.41	2	0.90	136.65	RM599	RM221
MQTL_2.5	2	0.1	38	25.35	148.04	RM6535	RM6424
MQTL_2.6	2	0.3	1	0.31	151.69	RM3857	RM573
MQTL_2.7	2	0.4	16	10.93	173.95	RM250	RM2265
MQTL_2.8	2	0.76	6	13.30	216.35	RM498	d29
MQTL_3.1	3	0.56	38	16.23	66.93	RM489	RG409
MQTL_3.2	3	4.7	12	20.07	85.85	RM3545	RM545
MQTL_3.3	3	2.42	7	18.10	115.44	RM5477	RM3872
MQTL_3.4	3	2.46	31	17.97	131.65	RM338	RM130
MQTL_3.5	3	1.69	19	28.14	212.75	RM6876	RM570
MQTL_4.1	4	0.27	23	15.33	38.95	RM6156	RM417
MQTL_4.2	4	0.39	17	17.10	62.46	RM5424	RM471
MQTL_4.3	4	0.31	2	8.04	76.93	RM119	RM3337
MQTL_4.4	4	0.43	7	14.71	90.13	RM1869	RM3866
MQTL_4.5	4	3.4	2	5.02	94.49	RM3839	RM1223
MQTL_4.6	4	1.56	5	6.96	100.89	RM3288	RM131
MQTL_4.7	4	0.06	8	14.39	105.15	RM2636	RM3276
MQTL_4.8	4	4.01	10	15.34	113.84	RM1153	RM303
MQTL_4.9	4	3.28	24	19.81	128.43	RM470	RM3648
MQTL_4.10	4	4.16	1	8.00	171.44	RM6246	RM280
MQTL_5.1	5	4.56	2	12.25	47.04	RM122	RM4777
MQTL_5.2	5	1.59	17	60.57	73.84	RM6229	RM507
MQTL_5.3	5	10.78	4	24.27	103.24	RM6082	RM163
MQTL_5.4	5	4.94	7	19.26	128.08	RM3351	RM440
MQTL_5.5	5	5.99	4	22.01	141.65	RM459	RM305
MQTL_5.6	5	4.27	11	21.60	156.66	RM173	RM6360
MQTL_5.7	5	1.3	3	15.78	172.99	RM6400	RM3790
MQTL_6.1	6	0.3	8	10.50	49.7	RM6775	RM4608
MQTL_6.2	6	1.36	8	12.92	57.57	RM6536	RM1163
MQTL_6.3	6	4.39	16	14.64	75.97	RM253	RM2126
MQTL_6.4	6	0.42	8	13.61	96.1	RM7488	RM6836
MQTL_6.5	6	0.44	4	10.89	111.31	RM527	RM564
MQTL_6.6	6	3.96	13	18.37	122.28	RM7583	RM3187
MQTL_6.7	6	6.36	7	19.34	152.74	RM275	RM5371
MQTL_6.8	6	1.75	9	21.47	191.95	RM5463	RM1150
MQTL_7.1	7	2.34	5	14.64	40.76	RM3224	RG528
MQTL_7.2	7	3.09	7	13.43	67.28	RM3186	RM8022
MQTL_7.3	7	3.4	5	10.18	72.89	RM8022	RM432
MQTL_7.4	7	0.68	7	24.99	92.48	RM560	RM3743
MQTL_7.5	7	7.56	2	11.25	106.72	RM5508	RM3583
MQTL_7.6	7	3.24	5	11.14	128.29	RM234	RM5720
MQTL_7.7	7	4	13	17.72	148.33	RM478	RM6650
MQTL_7.8	7	0.99	3	13.17	176.39	RM2789	RM248
MQTL_8.1	8	0.41	13	20.20	15.67	RM6925	RM2680
MQTL_8.2	8	6.03	8	17.76	52.34	RM5068	RM2584
MQTL_8.3	8	1.22	18	14.73	73.61	RM8243	RM1384
MQTL_8.4	8	4.03	4	7.93	90.21	RM3459	RM7356
MQTL_8.5	8	0.92	21	23.11	125.73	RM3754	RM3120
MQTL_9.1	9	2.45	23	11.15	84	RM316	RM5688
MQTL_9.2	9	0.1	65	15.10	130.37	RM5661	RZ228
MQTL_10.1	10	20.4	3	37.36	21.64	RM330	RM3229
MQTL_10.2	10	3.03	11	23.53	48.55	RM6207	RM5348
MQTL_10.3	10	8.19	1	8.90	60.2	RM6144	RM7300
MQTL_10.4	10	13.26	3	23.31	86.01	RM216	RM3451
MQTL_10.5	10	12.2	4	27.52	116.18	RM496	RM590
MQTL_11.1	11	10.24	10	36.02	41.22	RM3717	S20163S
MQTL_11.2	11	10.78	8	35.59	73.53	RM3701	RM457
MQTL_11.3	11	1.91	2	13.23	91.67	RM7391	RM5824
MQTL_11.4	11	1.92	8	15.91	104.69	RM209	RM5349
MQTL_11.5	11	0.64	15	23.32	108.33	RG2	RM206
MQTL_11.6	11	0.01	11	16.07	124.65	RM206	RM7170
MQTL_12.1	12	7.88	3	15.79	27.11	RM19	RM453
MQTL_12.2	12	13.04	2	18.78	52.26	RM1302	RM7195
MQTL_12.3	12	4.82	12	15.90	72.09	RM101	RM1261
MQTL_12.4	12	0.2	2	2.45	87.92	RM3331	RM6869
MQTL_12.5	12	10.6	2	15.50	100.06	RM6396	RM235
MQTL_12.6	12	1.46	11	16.58	117.49	RM1300	RM1310

## References

[1] Xing Y., Zhang Q. (2010). Genetic and Molecular Bases of Rice Yield. Annu. Rev. Plant Biol..

[2] Tester M., Langridge P. (2010). Breeding Technologies to Increase Crop Production in a Changing World. Science.

[3] Singhal A., Tien Y.-Y., Hsia R.Y. (2016). Racial-Ethnic Disparities in Opioid Prescriptions at Emergency Department Visits for Conditions Commonly Associated with Prescription Drug Abuse. PLoS ONE.

[4] Todaka D., Shinozaki K., Yamaguchi-Shinozaki K. (2015). Recent advances in the dissection of drought-stress regulatory networks and strategies for development of drought-tolerant transgenic rice plants. Front. Plant Sci..

[5] Panda D., Mishra S.S., Behera P.K. (2021). Drought Tolerance in Rice: Focus on Recent Mechanisms and Approaches. Rice Sci..

[6] Wang S., Yao Y., Wang J., Ruan B., Yu Y. (2025). Advancing Stress-Resilient Rice: Mechanisms, Genes, and Breeding Strategies. Agriculture.

[7] Blum A. (2011). Drought Resistance—Is It Really a Complex Trait?. Funct. Plant Biol..

[8] Zhu C., Ye Y., Qiu T., Huang Y., Ying J., Shen Z. (2024). Drought-Tolerant Rice at Molecular Breeding Eras: An Emerging Reality. Rice Sci..

[9] Sandhu N., Kumar A. (2017). Bridging the Rice Yield Gaps under Drought: QTLs, Genes, and their Use in Breeding Programs. Agronomy.

[10] Kaur S., Das A., Sheoran S., Rakshit S. (2023). QTL Meta-Analysis: An Approach to Detect Robust and Precise QTL. Trop. Plant Biol..

[11] Bilgrami S.S., Ramandi H.D., Shariati V., Razavi K., Tavakol E., Fakheri B.A., Nezhad N.M., Ghaderian M. (2020). Detection of genomic regions associated with tiller number in Iranian bread wheat under different water regimes using genome-wide association study. Sci. Rep..

[12] Swamy B.M., Vikram P., Dixit S., Ahmed H., Kumar A. (2011). Meta-analysis of grain yield QTL identified during agricultural drought in grasses showed consensus. BMC Genom..

[13] Saini D.K., Srivastava P., Pal N., Gupta P.K. (2022). Meta-QTLs, ortho-meta-QTLs and candidate genes for grain yield and associated traits in wheat (*Triticum aestivum* L.). Theor. Appl. Genet..

[14] Singh R.K., Kota S., Flowers T.J. (2021). Salt tolerance in rice: Seedling and reproductive stage QTL mapping come of age. Theor. Appl. Genet..

[15] Yu T., Zhang J., Cao J., Cao S., Li W., Yang G. (2022). A meta-analysis of low temperature tolerance QTL in maize. Electron. J. Biotechnol..

[16] Selamat N., Nadarajah K.K. (2021). Meta-Analysis of Quantitative Traits Loci (QTL) Identified in Drought Response in Rice (*Oryza sativa* L.). Plants.

[17] Aloryi K.D., Okpala N.E., Guo H., Karikari B., Amo A., Bello S.F., Saini D.K., Akaba S., Tian X. (2024). Integrated meta-analysis and transcriptomics pinpoint genomic loci and novel candidate genes associated with submergence tolerance in rice. BMC Genom..

[18] Kong W., Zhang C., Qiang Y., Zhong H., Zhao G., Li Y. (2020). Integrated RNA-seq Analysis and Meta-QTLs Mapping Provide Insights into Cold Stress Response in Rice Seedling Roots. Int. J. Mol. Sci..

[19] Yadaw R.B., Dixit S., Raman A., Mishra K.K., Vikram P., Swamy B.M., Cruz M.T.S., Maturan P.T., Pandey M., Kumar A. (2013). A QTL for high grain yield under lowland drought in the background of popular rice variety Sabitri from Nepal. Field Crop Res..

[20] Xing W., Zhao H., Zou D. (2014). Detection of main-effect and epistatic QTL for yield-related traits in rice under drought stress and normal conditions. Can. J. Plant Sci..

[21] Kim T.-H., Hur Y.-J., Han S.-I., Cho J.-H., Kim K.-M., Lee J.-H., Song Y.-C., Kwon Y.-U., Shin D. (2017). Drought-tolerant QTL qVDT11 leads to stable tiller formation under drought stress conditions in rice. Plant Sci..

[22] Baghyalakshmi K., Jeyaprakash P., Ramchander S., Raveendran M., Robin S. (2016). Fine mapping of rice drought QTL and study on combined effect of QTL for their physiological parameters under moisture stress condition. J. Appl. Nat. Sci..

[23] Sandhu N., Singh A., Dixit S., Cruz M.T.S., Maturan P.C., Jain R.K., Kumar A. (2014). Identification and mapping of stable QTL with main and epistasis effect on rice grain yield under upland drought stress. BMC Genet..

[24] Dixit S., Singh A., Cruz M.T.S., Maturan P.T., Amante M., Kumar A. (2014). Multiple major QTL lead to stable yield performance of rice cultivars across varying drought intensities. BMC Genet..

[25] Chen L., Ma J., Ma X., Cui D., Han B., Sun J., Han L. (2023). QTL analysis of drought tolerance traits in rice during the vegetative growth period. Euphytica.

[26] Songping H., Ying Z., Lin Z., Xudong Z., Zhenggong W., Lin L., Lijun L., Qingming Z. (2009). QTL analysis of floral traits of rice (*Oryza sativa* L.) under well-watered and drought stress conditions. Genes Genom..

[27] Barik S.R., Pandit E., Pradhan S.K., Singh S., Swain P., Mohapatra T. (2018). QTL Mapping for Relative Water Content Trait at Re-productive Stage Drought Stress in Rice. Indian J. Genet..

[28] Mardani Z., Rabiei B., Sabouri H., Sabouri A. (2013). Mapping of QTLs for Germination Characteristics under Non-stress and Drought Stress in Rice. Rice Sci..

[29] Liu G., Mei H., Yu X., Zou G., Liu H., Hu S., Li M., Wu J., Chen L., Luo L. (2008). QTL analysis of panicle neck diameter, a trait highly correlated with panicle size, under well-watered and drought conditions in rice (*Oryza sativa* L.). Plant Sci..

[30] Sharma V., Verma R.K., Dey P., Chetia S., Baruah A., Modi M. (2017). QTLs associated with yield attributing traits under drought stress in upland rice cultivar of Assam. Oryza-An Int. J. Rice.

[31] Chen M.-Y., Ali J., Fu B.-Y., Xu J.-L., Zhao M.-F., Jiang Y.-Z., Zhu L.-H., Shi Y.-Y., Yao D.-N., Gao Y.-M. (2011). Detection of Drought-Related Loci in Rice at Reproductive Stage Using Selected Introgressed Lines. Agric. Sci. China.

[32] Hu S.-P., Yang H., Zou G.-H., Liu H.-Y., Liu G.-L., Mei H.-W., Cai R., Li M.-S., Luo L.-J. (2007). Relationship Between Coleoptile Length and Drought Resistance and Their QTL Mapping in Rice. Rice Sci..

[33] Manickavelu A., Nadarajan N., Ganesh S.K., Gnanamalar R.P., Babu R.C. (2006). Drought tolerance in rice: Morphological and molecular genetic consideration. Plant Growth Regul..

[34] Saikumar S., Gouda P.K., Saiharini A., Varma C.M.K., Vineesha O., Padmavathi G., Shenoy V. (2014). Major QTL for enhancing rice grain yield under lowland reproductive drought stress identified using an *O. sativa*/*O. glaberrima* introgression line. Field Crop Res..

[35] Barik S., Pandit E., Mohanty S., Pradhan S. (2018). QTL mapping for traits at reproductive stage drought stress in rice using single marker analysis. Oryza-An Int. J. Rice.

[36] Sellamuthu R., Ranganathan C., Serraj R. (2015). Mapping QTLs for Reproductive-Stage Drought Resistance Traits using an Advanced Backcross Population in Upland Rice. Crop Sci..

[37] Nguyen T.T.T., Klueva N., Chamareck V., Aarti A., Magpantay G., Millena A.C.M., Pathan M.S., Nguyen H.T. (2004). Saturation mapping of QTL regions and identification of putative candidate genes for drought tolerance in rice. Mol. Genet. Genom..

[38] Liu G., Mei H., Liu H., Yu X., Zou G., Luo L. (2009). Sensitivities of rice grain yield and other panicle characters to late-stage drought stress revealed by phenotypic correlation and QTL analysis. Mol. Breed..

[39] Ali M.L., Pathan M.S., Zhang J., Bai G., Sarkarung S., Nguyen H.T. (2000). Mapping QTLs for root traits in a recombinant inbred population from two indica ecotypes in rice. Theor. Appl. Genet..

[40] Bernier J., Kumar A., Ramaiah V., Spaner D., Atlin G. (2007). A Large-Effect QTL for Grain Yield under Reproductive-Stage Drought Stress in Upland Rice. Crop Sci..

[41] Courtois B., Shen L., Petalcorin W., Carandang S., Mauleon R., Li Z. (2003). Locating QTLs controlling constitutive root traits in the rice population IAC 165 × Co39. Euphytica.

[42] Gu J., Yin X., Struik P.C., Stomph T.J., Wang H. (2011). Using chromosome introgression lines to map quantitative trait loci for photosynthesis parameters in rice (*Oryza sativa* L.) leaves under drought and well-watered field conditions. J. Exp. Bot..

[43] Hemamalini G., Shashidhar H., Hittalmani S. (2000). Molecular marker assisted tagging of morphological and physiological traits under two contrasting moisture regimes at peak vegetative stage in rice (*Oryza sativa* L.). Euphytica.

[44] Horii H., Nemoto K., Miyamoto N., Harada J. (2006). Quantitative trait loci for adventitious and lateral roots in rice. Plant Breed..

[45] Kamoshita A., Wade L., Ali M., Pathan M., Zhang J., Sarkarung S., Nguyen H. (2002). Mapping QTLs for root morphology of a rice population adapted to rainfed lowland conditions. Theor. Appl. Genet..

[46] Kamoshita A., Zhang J., Siopongco J., Sarkarung S., Nguyen H.T., Wade L.J. (2002). Effects of Phenotyping Environment on Iden-tification of Quantitative Trait Loci for Rice Root Morphology under Anaerobic Conditions. Crop Sci..

[47] Kato Y., Hirotsu S., Nemoto K., Yamagishi J. (2007). Identification of QTLs controlling rice drought tolerance at seedling stage in hydroponic culture. Euphytica.

[48] Kumar R., Venuprasad R., Atlin G. (2007). Genetic analysis of rainfed lowland rice drought tolerance under naturally-occurring stress in eastern India: Heritability and QTL effects. Field Crop Res..

[49] Lanceras J.C., Pantuwan G., Jongdee B., Toojinda T. (2004). Quantitative Trait Loci Associated with Drought Tolerance at Reproductive Stage in Rice. Plant Physiol..

[50] Li Z., Mu P., Li C., Zhang H., Li Z., Gao Y., Wang X. (2005). QTL mapping of root traits in a doubled haploid population from a cross between upland and lowland japonica rice in three environments. Theor. Appl. Genet..

[51] MacMillan K., Emrich K., Piepho H.-P., Mullins C.E., Price A.H. (2006). Assessing the importance of genotype × environment interaction for root traits in rice using a mapping population II: Conventional QTL analysis. Theor. Appl. Genet..

[52] Gomez S.M., Boopathi N.M., Kumar S.S., Ramasubramanian T., Chengsong Z., Jeyaprakash P., Senthil A., Babu R.C. (2009). Molecular mapping and location of QTLs for drought-resistance traits in indica rice (*Oryza sativa* L.) lines adapted to target environments. Acta Physiol. Plant..

[53] Palanog A.D., Swamy B.M., Shamsudin N.A.A., Dixit S., Hernandez J.E., Boromeo T.H., Cruz P.C.S., Kumar A. (2014). Grain yield QTLs with consistent-effect under reproductive-stage drought stress in rice. Field Crop Res..

[54] Price A.H., Steele K.A., Moore B.J., Barraclough P.P., Clark L.J. (2000). A combined RFLP and AFLP linkage map of upland rice (*Oryza sativa* L.) used to identify QTLs for root-penetration ability. Theor. Appl. Genet..

[55] Price A.H., Steele K.A., Moore B.J., Jones R.G.W. (2002). Upland Rice Grown in Soil-®lled Chambers and Exposed to Contrasting Water-De®cit Regimes II. Mapping Quantitative Trait Loci for Root Morphology and Distribution. Field Crops Res..

[56] Prince S.J., Beena R., Gomez S.M., Senthivel S., Babu R.C. (2015). Mapping Consistent Rice (*Oryza sativa* L.) Yield QTLs under Drought Stress in Target Rainfed Environments. Rice.

[57] Redofia E.D., Mackill D.J. (1996). Mapping Quantitative Trait Loci for Seedling Vigor in Rice Using RFLPs. Theor. Appl. Genet..

[58] Sabar M., Shabir G., Shah S.M., Aslam K., Naveed S.A., Arif M. (2019). Identification and mapping of QTLs associated with drought tolerance traits in rice by a cross between Super Basmati and IR55419-04. Breed. Sci..

[59] Sandhu N., Jain S., Kumar A., Mehla B.S., Jain R. (2013). Genetic variation, linkage mapping of QTL and correlation studies for yield, root, and agronomic traits for aerobic adaptation. BMC Genet..

[60] Shen L., Courtois B., McNally K.L., Robin S., Li Z. (2001). Evaluation of near-isogenic lines of rice introgressed with QTLs for root depth through marker-aided selection. Theor. Appl. Genet..

[61] Solis J., Gutierrez A., Mangu V., Sanchez E., Bedre R., Linscombe S., Baisakh N. (2018). Genetic Mapping of Quantitative Trait Loci for Grain Yield under Drought in Rice under Controlled Greenhouse Conditions. Front. Chem..

[62] Tripathy J.N., Zhang J., Robin S., Nguyen T.T., Nguyen H.T. (2000). QTLs for cell-membrane stability mapped in rice (*Oryza sativa* L.) under drought stress. Theor. Appl. Genet..

[63] Xu C.G., Li X.Q., Xue Y., Huang Y.W., Gao J., Xing Y.Z. (2004). Comparison of quantitative trait loci controlling seedling characteristics at two seedling stages using rice recombinant inbred lines. Theor. Appl. Genet..

[64] Yue B., Xue W., Xiong L., Yu X., Luo L., Cui K., Jin D., Xing Y., Zhang Q. (2006). Genetic Basis of Drought Resistance at Reproductive Stage in Rice: Separation of Drought Tolerance from Drought Avoidance. Genetics.

[65] You J., Li Q., Yue B., Xue W.-Y., Luo L.-J., Xiong L.-Z. (2006). Identification of Quantitative Trait Loci for ABA Sensitivity at Seed Germination and Seedling Stages in Rice. Acta Genet. Sin..

[66] Yue B., Xue W., Luo L., Xing Y. (2008). Identification of quantitative trait loci for four morphologic traits under water stress in rice (*Oryza sativa* L.). J. Genet. Genom..

[67] Yue B., Xiong L., Xue W., Xing Y., Luo L., Xu C. (2005). Genetic analysis for drought resistance of rice at reproductive stage in field with different types of soil. Theor. Appl. Genet..

[68] Zhang J., Zheng H.G., Aarti A., Pantuwan G., Nguyen T.T., Tripathy J.N., Sarial A.K., Robin S., Babu R.C., Nguyen B.D. (2001). Locating genomic regions associated with components of drought resistance in rice: Comparative mapping within and across species. Theor. Appl. Genet..

[69] Zheng B.S., Yang L., Zhang W.P., Mao C.Z., Wu Y.R., Yi K.K., Liu F.Y., Wu P. (2003). Mapping QTLs and candidate genes for rice root traits under different water-supply conditions and comparative analysis across three populations. Theor. Appl. Genet..

[70] Zheng H., Babu M.R.C., Pathan M.S., Ali L., Huang N., Courtois B., Nguyen H.T. (2000). Quantitative Trait Loci for Root-Penetration Ability and Root Thickness in Rice: Comparison of Genetic Backgrounds. Genome.

[71] Zheng B., Yang L., Mao C., Huang Y., Wu P. (2008). Comparison of QTLs for rice seedling morphology under different water supply conditions. J. Genet. Genom..

[72] Liu T., Li S., Du H., Cui J., Xu S., Wang J., Liu H., Zou D., Lu W., Zheng H. (2024). The Identification of Drought Tolerance Candidate Genes in *Oryza Sativa* L. Ssp. Japonica Seedlings through Genome-Wide Association Study and Linkage Mapping. Agriculture.

[73] Kadam N.N., Struik P.C., Rebolledo M.C., Yin X., Jagadish S.V.K. (2018). Genome-Wide Association Reveals Novel Genomic Loci Controlling Rice Grain Yield and Its Component Traits under Water-Deficit Stress during the Reproductive Stage. J. Exp. Bot..

[74] Bhandari A., Sandhu N., Bartholome J., Cao-Hamadoun T.-V., Ahmadi N., Kumari N., Kumar A. (2020). Genome-Wide Association Study for Yield and Yield Related Traits under Reproductive Stage Drought in a Diverse Indica-Aus Rice Panel. Rice.

[75] Muthukumar C., Subathra T., Aiswarya J., Gayathri V., Babu R.C. (2015). Comparative Genome-Wide Association Studies for Plant Production Traits under Drought in Diverse Rice (*Oryza Sativa* L.) Lines Using SNP and SSR Markers. CURRENT SCIENCE.

[76] Sakhare A.S., Kumar S., Ellur R.K., Prahalada G.D., Kota S., Kumar R.R., Ray S., Mandal B.N., Chinnusamy V. (2025). Genome-Wide Association Study on Root Traits under Non-Stress and Osmotic Stress Conditions to Improve Drought Tolerance in Rice (*Oryza Sativa* Lin.). Acta Physiol. Plant.

[77] Wang N., Gao Z., Zhang W., Qian Y., Bai D., Zhao X., Bao Y., Zheng Z., Wang X., Li J. (2023). Genome-Wide Association Analysis Reveals the Gene Loci of Yield Traits under Drought Stress at the Rice Reproductive Stage. Agronomy.

[78] Yi Y., Hassan M.A., Cheng X., Li Y., Liu H., Fang W., Zhu Q., Wang S. (2023). QTL Mapping and Analysis for Drought Tolerance in Rice by Genome-Wide Association Study. Front. Plant Sci..

[79] Gu J., Peng X., Guo S., Lu J., Shi X., Sun Y., Yang J. (2025). Innovation and Implement of Green Technology in Rice Production to Increase Yield and Resource Use Efficiency. Front. Agric. Sci. Eng..

[80] Hua J., Xing Y., Wu W., Xu C., Sun X., Yu S., Zhang Q. (2003). Single-locus heterotic effects and dominance by dominance interactions can adequately explain the genetic basis of heterosis in an elite rice hybrid. Proc. Natl. Acad. Sci. USA.

[81] Guo B., Sleper D.A., Lu P., Shannon J.G., Nguyen H.T., Arelli P.R. (2006). QTLs Associated with Resistance to Soybean Cyst Nematode in Soybean: Meta-Analysis of QTL Locations. Crop Sci..

[82] Loni F., Ismaili A., Nakhoda B., Ramandi H.D., Shobbar Z.-S. (2023). The genomic regions and candidate genes associated with drought tolerance and yield-related traits in foxtail millet: An integrative meta-analysis approach. Plant Growth Regul..

[83] Eweda M.A., Yabes J. (2025). Molecular and Physiological Characterizations of Roots under Drought Stress in Rice: A Comprehensive Review. Plant Physiol. Biochem..

[84] Goyal S., Sandhu D. (2024). Defining Genomic Landscape for Identification of Potential Candidate Resistance Genes Associated with Major Rice Diseases through MetaQTL Analysis. J. Biosci..

[85] Devanna B.N., Sucharita S., Sunitha N.C., Anilkumar C., Singh P.K., Pramesh D., Samantaray S., Behera L., Katara J.L., Parameswaran C. (2024). Refinement of rice blast disease resistance QTLs and gene networks through meta-QTL analysis. Sci. Rep..

[86] Khahani B., Taghavi E. (2011). Meta-QTL and Ortho-MQTL Analyses Identified Genomic Regions Controlling Rice Yield, Yield-Related Traits and Root Architecture under Water Deficit Conditions. Sci. Rep..

[87] Daryani P., Amirbakhtiar N., Soorni J., Loni F., Ramandi H.D., Shobbar Z.-S. (2024). Uncovering the Genomic Regions Associated with Yield Maintenance in Rice Under Drought Stress Using an Integrated Meta-Analysis Approach. Rice.

[88] Cui Y., Zhang W., Lin X., Xu S., Xu J., Li Z. (2018). Simultaneous Improvement and Genetic Dissection of Drought Tolerance Using Selected Breeding Populations of Rice. Front. Plant Sci..

[89] Zhang Y., Lin X.-F., Li L., Piao R.-H., Wu S., Song A., Gao M., Jin Y.-M. (2024). CRISPR/Cas9-mediated knockout of Bsr-d1 enhances the blast resistance of rice in Northeast China. Plant Cell Rep..

[90] Ma T.-C., Chen R.-J., Yu R.-R., Zeng H.-L., Zhang D.-P. (2009). Differential global genomic changes in rice root in response to low-, middle-, and high-osmotic stresses. Acta Physiol. Plant..

[91] Yan S., Liu X., Huang Y., Ma Z., Wang W., Li N., Liu Z., Yang H., Zhang X. (2024). The Effect of Simulated Drought on Root System in Rice Cultivars with Differential Drought Stress Tolerance. Mol. Plant Breed..

[92] Ma X., Xia H., Liu Y., Wei H., Zheng X., Song C., Chen L., Liu H., Luo L. (2016). Transcriptomic and Metabolomic Studies Disclose Key Metabolism Pathways Contributing to Well-maintained Photosynthesis under the Drought and the Consequent Drought-Tolerance in Rice. Front. Plant Sci..

[93] Kim Y., Chung Y.S., Lee E., Tripathi P., Heo S., Kim K.-H. (2020). Root Response to Drought Stress in Rice (*Oryza sativa* L.). Int. J. Mol. Sci..

[94] Fonta J.E., Giri J., Vejchasarn P., Lynch J.P., Brown K.M. (2022). Spatiotemporal responses of rice root architecture and anatomy to drought. Plant Soil.

[95] Xiong H., Yu J., Miao J., Li J., Zhang H., Wang X., Liu P., Zhao Y., Jiang C., Yin Z. (2018). Natural Variation in *OsLG3* Increases Drought Tolerance in Rice by Inducing ROS Scavenging. Plant Physiol..

[96] Nadarajah K.K. (2020). ROS Homeostasis in Abiotic Stress Tolerance in Plants. Int. J. Mol. Sci..

[97] Xie X., He Z., Chen N., Tang Z., Wang Q., Cai Y. (2019). The Roles of Environmental Factors in Regulation of Oxidative Stress in Plant. BioMed Res. Int..

[98] Luo H., Wu B., Amin B., Li J., Chen Z., Shi J., Huang W., Fang Z. (2025). Amino Acid Regulation in Rice: Integrated Mechanisms and Agricultural Applications. Rice.

[99] Babitha M.P., Bhat S.G., Prakash H.S., Shetty H.S. (2002). Differential induction of superoxide dismutase in downy mildew-resistant and -susceptible genotypes of pearl millet. Plant Pathol..

[100] Li Q., Zhu P., Yu X., Xu J., Liu G. (2024). Physiological and Molecular Mechanisms of Rice Tolerance to Salt and Drought Stress: Advances and Future Directions. Int. J. Mol. Sci..

[101] Liu T., Liu L., Zhou T., Chen Y., Zhou H., Lyu J., Zhang D., Shi X., Yuan D., Ye N. (2025). Chalcone isomerase gene (OsCHI3) increases rice drought tolerance by scavenging ROS via flavonoid and ABA metabolic pathways. Crop J..

[102] Dossa G.S., Torres R., Henry A., Oliva R., Maiss E., Cruz C.V., Wydra K. (2016). Rice response to simultaneous bacterial blight and drought stress during compatible and incompatible interactions. Eur. J. Plant Pathol..

[103] Tao Z., Zhu L., Li H., Sun B., Liu X., Li D., Hu W., Wang S., Miao X., Shi Z. (2024). ACL1-ROC4/5 complex reveals a common mechanism in rice response to brown planthopper infestation and drought. Nat. Commun..

[104] Ghorbanzadeh Z., Hamid R., Jacob F., Zeinalabedini M., Salekdeh G.H., Ghaffari M.R. (2023). Comparative metabolomics of root-tips reveals distinct metabolic pathways conferring drought tolerance in contrasting genotypes of rice. BMC Genom..

[105] Li C., Fu K., Guo W., Zhang X., Li C., Li C. (2023). Starch and Sugar Metabolism Response to Post-Anthesis Drought Stress During Critical Periods of Elite Wheat (*Triticum aestivum* L.) Endosperm Development. J. Plant Growth Regul..

[106] Liao Z., Zhang Y., Yu Q., Fang W., Chen M., Li T., Liu Y., Liu Z., Chen L., Yu S. (2023). Co-ordination of Growth and Drought Responses by GA-ABA Signaling in Rice. New Phytol..

[107] Auler P.A., Souza G.M., Engela M.R.G.d.S., Amaral M.N.D., Rossatto T., da Silva M.G.Z., Furlan C.M., Maserti B., Braga E.J.B. (2021). Stress memory of physiological, biochemical and metabolomic responses in two different rice genotypes under drought stress: The scale matters. Plant Sci..

[108] Zhou H., Zhang F., Zhai F., Su Y., Zhou Y., Ge Z., Tilak P., Eirich J., Finkemeier I., Fu L. (2022). Rice GLUTATHIONE PEROXIDASE1-mediated oxidation of bZIP68 positively regulates ABA-independent osmotic stress signaling. Mol. Plant.

[109] Muthuramalingam P., Krishnan S.R., Pothiraj R., Ramesh M. (2017). Global Transcriptome Analysis of Combined Abiotic Stress Signaling Genes Unravels Key Players in *Oryza sativa* L.: An In silico Approach. Front. Plant Sci..

[110] Bashyal B.M., Parmar P., Zaidi N.W., Aggarwal R. (2021). Molecular Programming of Drought-Challenged *Trichoderma harzianum*-Bioprimed Rice (*Oryza sativa* L.). Front. Microbiol..

[111] Kosugi T., Ohue M. (2023). Design of Cyclic Peptides Targeting Protein–Protein Interactions Using AlphaFold. Int. J. Mol. Sci..

[112] Zhang G., Zhang C., Cai M., Luo C., Zhu F., Liang Z. (2024). FuncPhos-STR: An integrated deep neural network for functional phosphosite prediction based on AlphaFold protein structure and dynamics. Int. J. Biol. Macromol..

[113] Darvasi A., Soller M. (1997). A Simple Method to Calculate Resolving Power and Confidence Interval of QTL Map Location. Behav. Genet..

[114] Sosnowski O., Charcosset A., Joets J. (2012). BioMercator V3: An upgrade of genetic map compilation and quantitative trait loci meta-analysis algorithms. Bioinformatics.

[115] Arcade A., Labourdette A., Falque M., Mangin B., Chardon F., Charcosset A., Joets J. (2004). BioMercator: Integrating genetic maps and QTL towards discovery of candidate genes. Bioinformatics.

[116] Chardon F., Virlon B., Moreau L., Falque M., Joets J., Decousset L., Murigneux A., Charcosset A. (2004). Genetic Architecture of Flowering Time in Maize as Inferred from Quantitative Trait Loci Meta-analysis and Synteny Conservation with the Rice Genome. Genetics.

[117] Daryani P., Ramandi H.D., Dezhsetan S., Mansuri R.M., Salekdeh G.H., Shobbar Z.-S. (2021). Pinpointing genomic regions associated with root system architecture in rice through an integrative meta-analysis approach. Theor. Appl. Genet..

