# Supplementary material for: An Integrated Meta-QTL and Transcriptome Analysis Provides Candidate Genes Associated with Drought Tolerance in Rice Seedlings
Source: Plants (Basel). 2025 Nov 29;14(23):3645. doi: 10.3390/plants14233645 (PMC12693912; doi:10.3390/plants14233645)
Supplement: Supplementary file 1 [file plants-14-03645-s001.zip › Supplementary Figure S1.pdf]

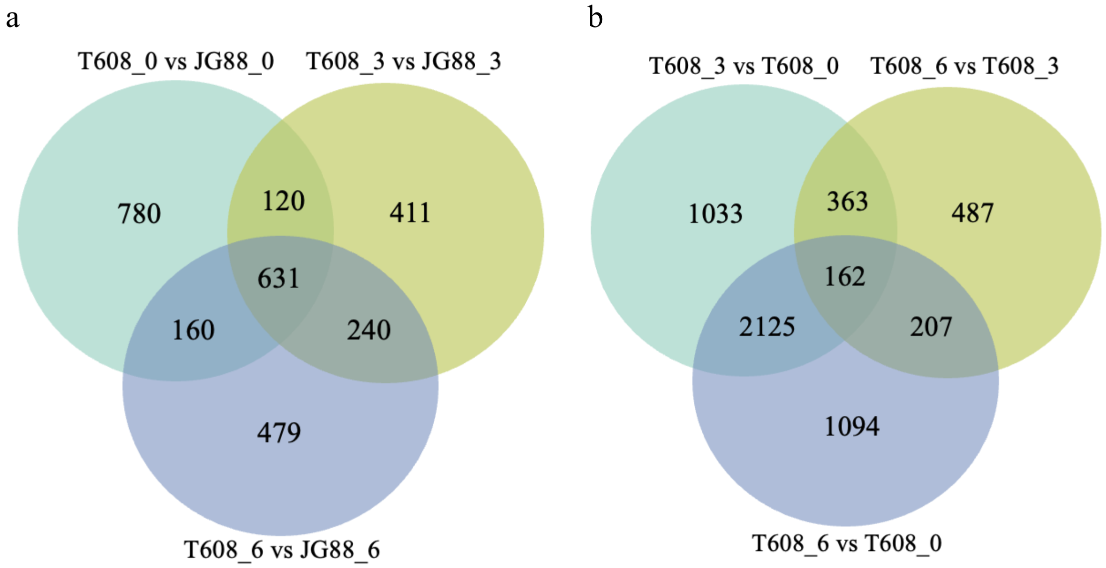

Figure S1: Distribution of drought-responsive DEGs between JG88 and T608. (a) DEGs identified in pairwise comparisons of T608 vs. JG88 at three independent time points. (b) DEGs identified in pairwise comparisons among T608 samples collected at different time points.
