# Supplementary material for: An Integrated Meta-QTL and Transcriptome Analysis Provides Candidate Genes Associated with Drought Tolerance in Rice Seedlings
Source: Plants (Basel). 2025 Nov 29;14(23):3645. doi: 10.3390/plants14233645 (PMC12693912; doi:10.3390/plants14233645)
Supplement: Supplementary file 1 [file plants-14-03645-s001.zip › Supplementary Figure S2.pdf]

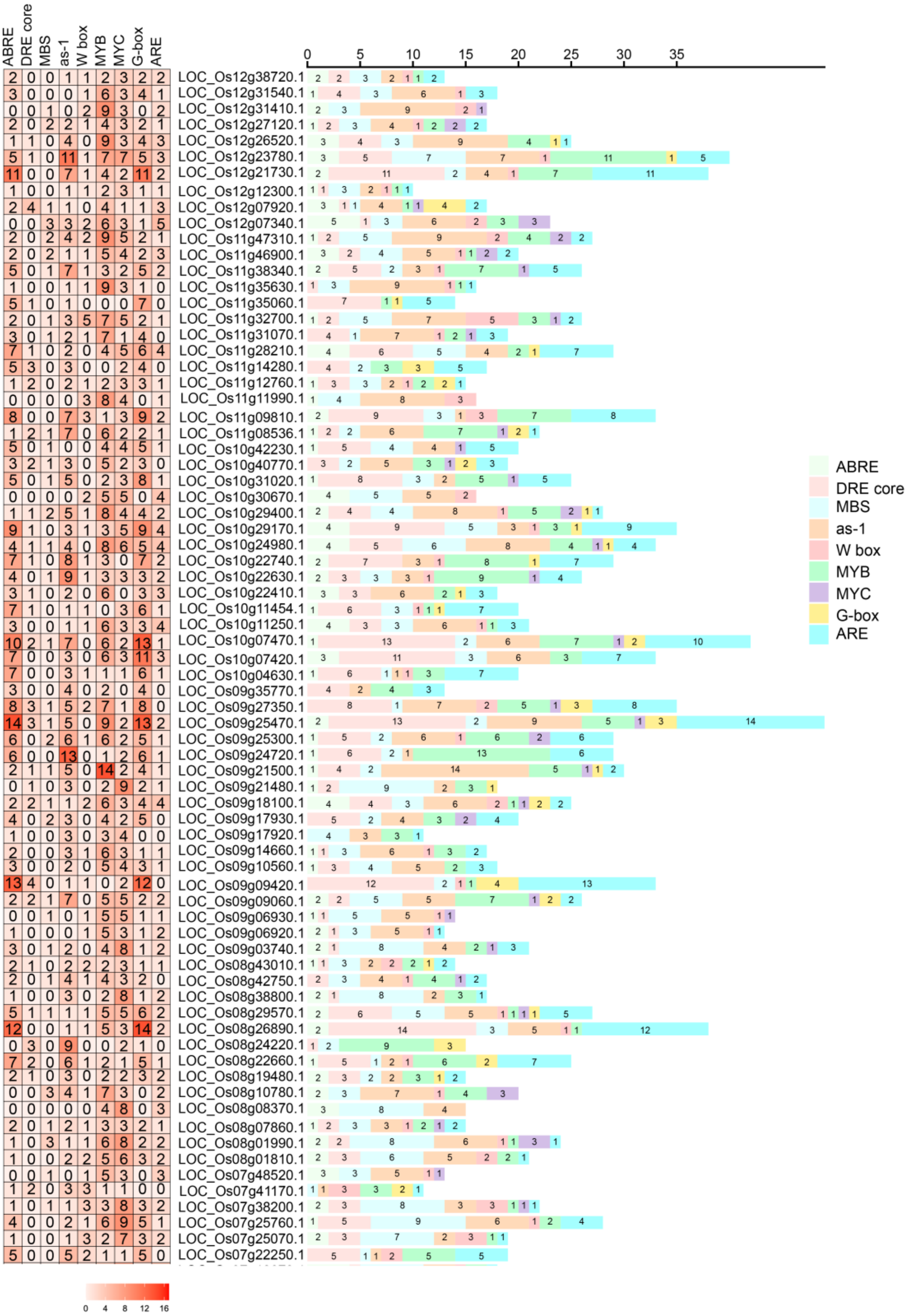
ABRE
DRE core
MBS
as-1
W box
MYB
MYC
G-box
ARE
0
5
10
15
20
25
30
35
ABRE
DRE core
MBS
as-1
W box
MYB
MYC
G-box
ARE
0
4
8
12
16

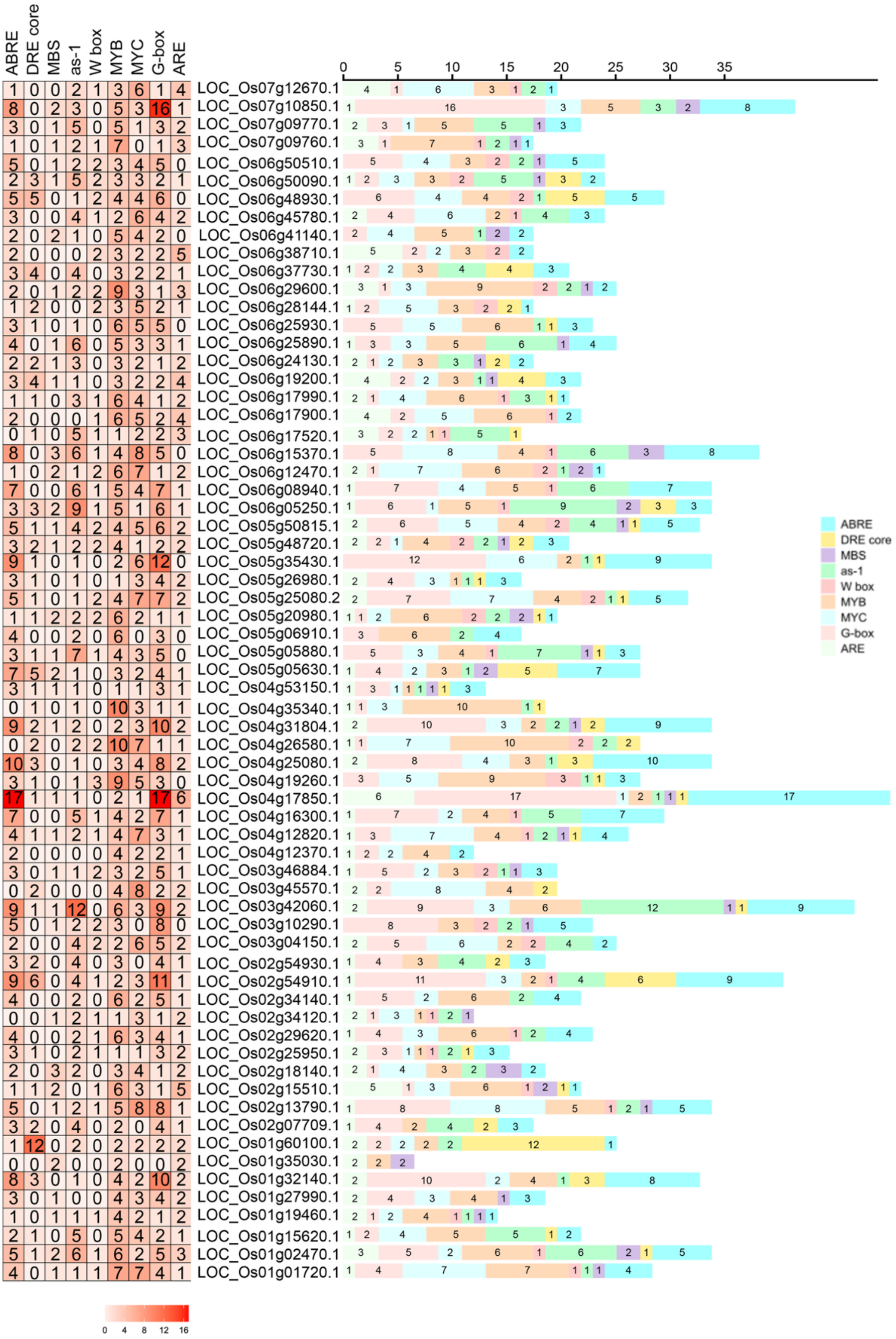
ABRE
DRE core
MBS
as-1
W box
MYB
MYC
G-box
ARE
0
5
10
15
20
25
30
35
LOC_Os07g12670.1
LOC_Os07g10850.1
LOC_Os07g09770.1
LOC_Os07g09760.1
LOC_Os06g50510.1
LOC_Os06g50090.1
LOC_Os06g48930.1
LOC_Os06g45780.1
LOC_Os06g41140.1
LOC_Os06g38710.1
LOC_Os06g37730.1
LOC_Os06g29600.1
LOC_Os06g28144.1
LOC_Os06g25930.1
LOC_Os06g25890.1
LOC_Os06g24130.1
LOC_Os06g19200.1
LOC_Os06g17990.1
LOC_Os06g17900.1
LOC_Os06g17520.1
LOC_Os06g15370.1
LOC_Os06g12470.1
LOC_Os06g08940.1
LOC_Os06g05250.1
LOC_Os05g50815.1
LOC_Os05g48720.1
LOC_Os05g35430.1
LOC_Os05g26980.1
LOC_Os05g25080.2
LOC_Os05g20980.1
LOC_Os05g06910.1
LOC_Os05g05880.1
LOC_Os05g05630.1
LOC_Os04g53150.1
LOC_Os04g35340.1
LOC_Os04g31804.1
LOC_Os04g26580.1
LOC_Os04g25080.1
LOC_Os04g19260.1
LOC_Os04g17850.1
LOC_Os04g16300.1
LOC_Os04g12820.1
LOC_Os04g12370.1
LOC_Os03g46884.1
LOC_Os03g45570.1
LOC_Os03g42060.1
LOC_Os03g10290.1
LOC_Os03g04150.1
LOC_Os02g54930.1
LOC_Os02g54910.1
LOC_Os02g34140.1
LOC_Os02g34120.1
LOC_Os02g29620.1
LOC_Os02g25950.1
LOC_Os02g18140.1
LOC_Os02g15510.1
LOC_Os02g13790.1
LOC_Os02g07709.1
LOC_Os01g60100.1
LOC_Os01g35030.1
LOC_Os01g32140.1
LOC_Os01g27990.1
LOC_Os01g19460.1
LOC_Os01g15620.1
LOC_Os01g02470.1
LOC_Os01g01720.1
ABRE
DRE core
MBS
as-1
W box
MYB
MYC
G-box
ARE
0
4
8
12
16

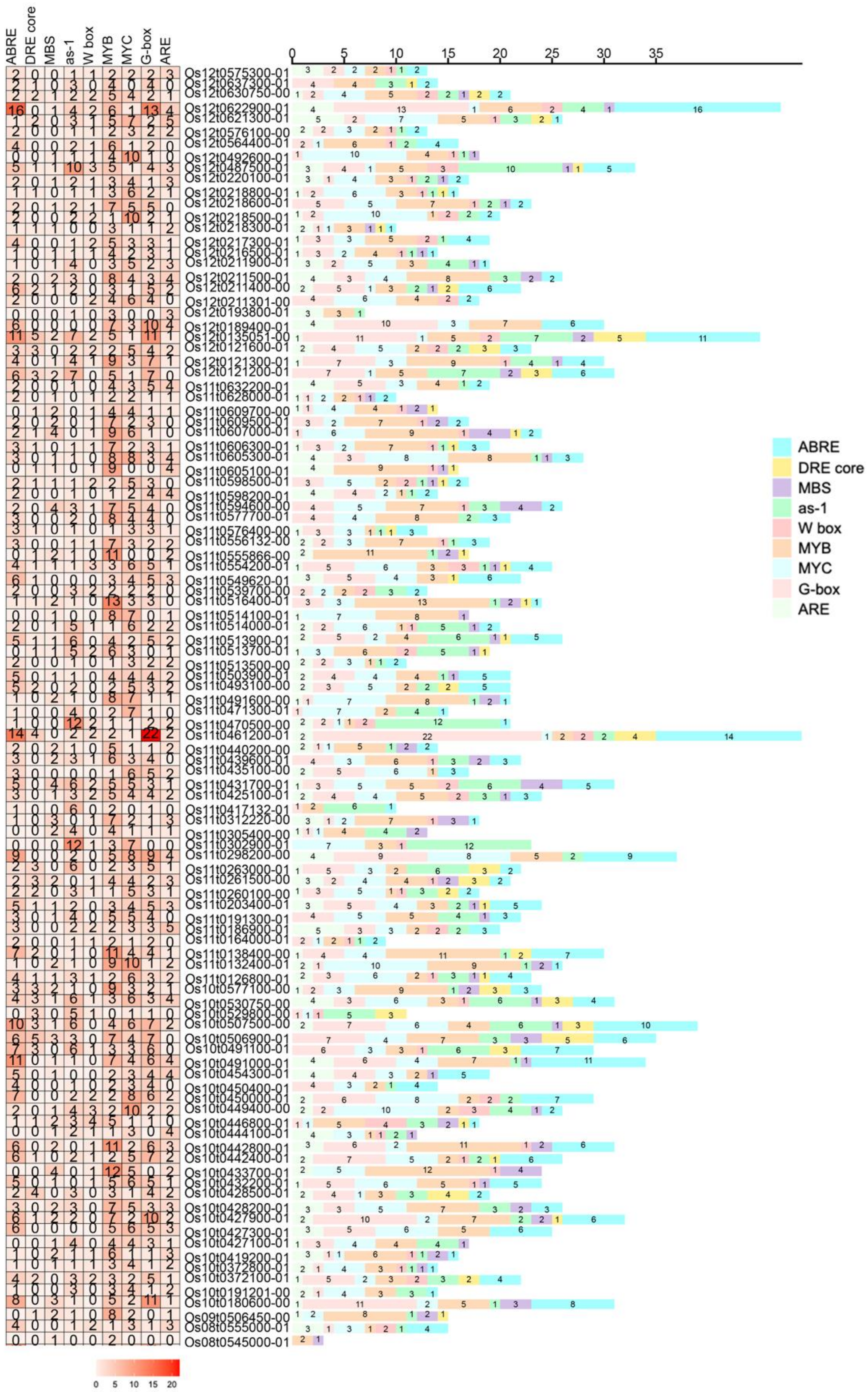
ABRE
DRE core
MBS
as-1
W box
MYB
MYC
G-box
ARE
0
5
10
15
20
25
30
35
ABRE
DRE core
MBS
as-1
W box
MYB
MYC
G-box
ARE
0
5
10
15
20

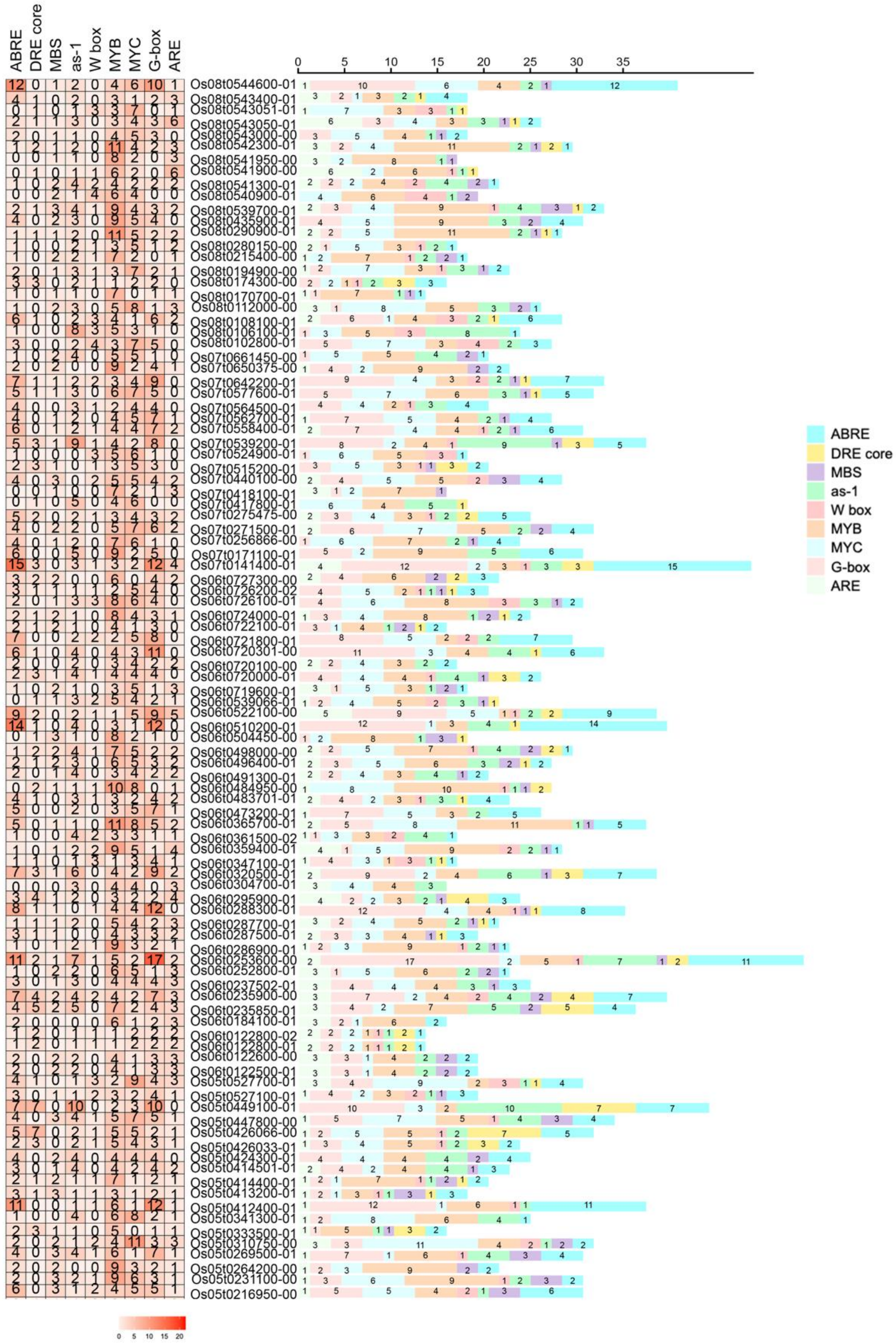

ABRE
DRE core
MBS
as-1
W box
MYB
MYC
G-box
ARE
0
5
10
15
20
25
30
35
ABRE
DRE core
MBS
as-1
W box
MYB
MYC
G-box
ARE
0 5 10 15 20

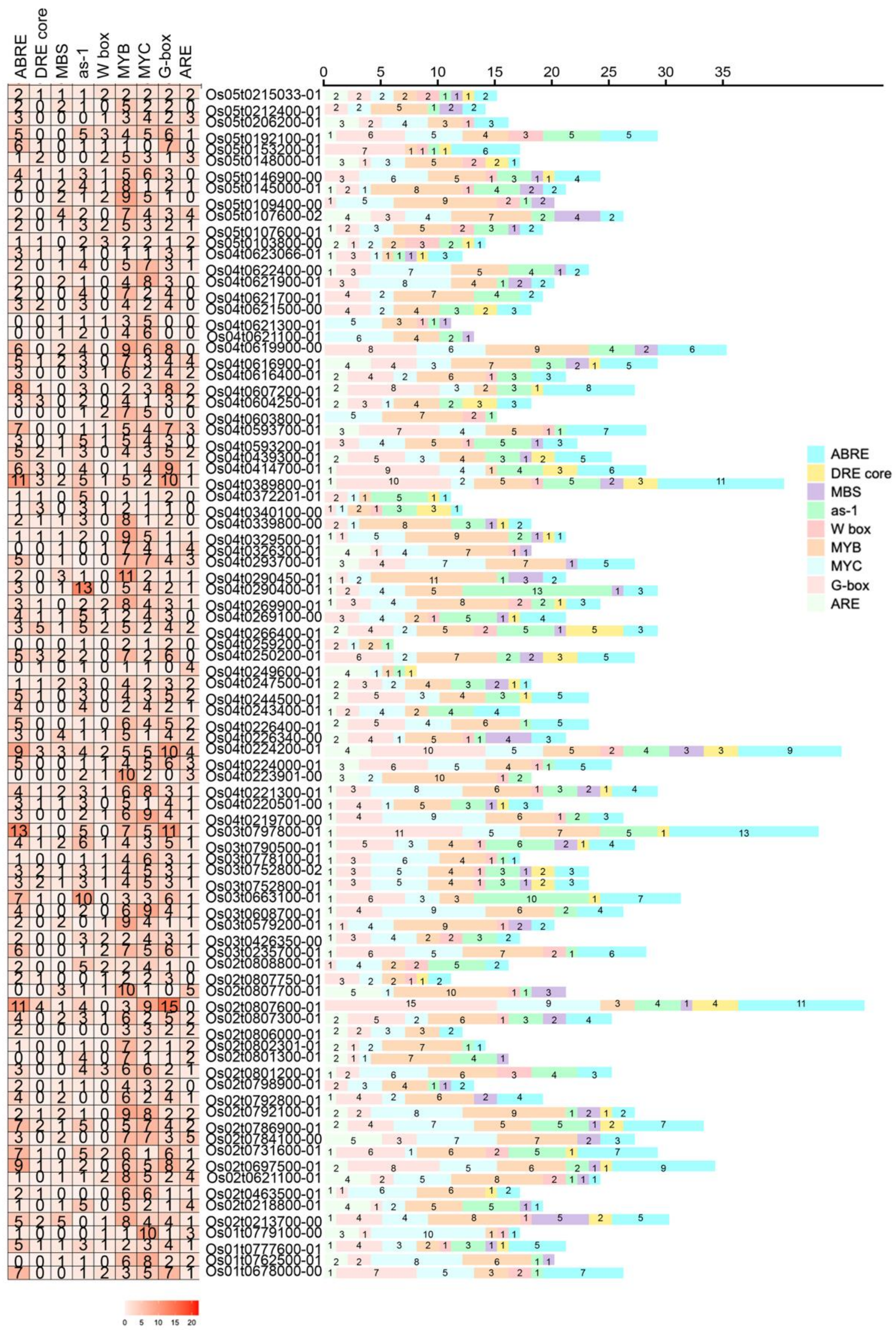

Figure S2: The heatmap depicts the abundance profile of core CREs in the promoters of drought-responsive DECGs, showcasing the variation in their copy numbers.
